# R-Ras subfamily proteins elicit distinct physiologic effects and phosphoproteome alterations in neurofibromin-null MPNST cells

**DOI:** 10.1186/s12964-021-00773-4

**Published:** 2021-09-16

**Authors:** Shannon M. Weber, Nicole M. Brossier, Amanda Prechtl, Stephen Barnes, Landon S. Wilson, Stephanie N. Brosius, Jody Fromm Longo, Steven L. Carroll

**Affiliations:** 1 Department of Pathology and Laboratory Medicine (SMW, AP, JFL, SLC), MUSC Medical Scientist Training Program (SMW), Medical University of South Carolina, 171 Ashley Avenue, MSC 908, Charleston, SC 29425-9080 USA; 2 Departments of Pathology (NMB, SNB, SLC), Pharmacology and Toxicology (SB, LSW), UAB Medical Scientist Training Program (NMB, SNB), Birmingham, USA; 3 The University of Alabama at Birmingham, Birmingham, AL 35294 USA; 4 Present Address: Department of Pediatrics, St. Louis Children’s Hospital, St. Louis, USA; 5 Present Address: Departments of Neurology and Pediatrics, Perelman School of Medicine, University of Pennsylvania, Philadelphia, USA; 6 Present Address: Division of Child Neurology, Children’s Hospital of Philadelphia, Philadelphia, USA

**Keywords:** Neurofibromatosis, Malignant peripheral nerve sheath tumor, Ras, R-Ras, TC21

## Abstract

**Background:**

Loss of the Ras GTPase-activating protein neurofibromin promotes nervous system tumor pathogenesis in patients with neurofibromatosis type 1 (NF1). Neurofibromin loss potentially hyperactivates classic Ras (H-Ras, N-Ras, K-Ras), M-Ras, and R-Ras (R-Ras, R-Ras2/TC21) subfamily proteins. We have shown that classic Ras proteins promote proliferation and survival, but not migration, in malignant peripheral nerve sheath tumor (MPNST) cells. However, it is unclear whether R-Ras, R-Ras2 and M-Ras are expressed and hyperactivated in MPNSTs and, if so, whether they contribute to MPNST pathogenesis. We assessed the expression and activation of these proteins in MPNST cells and inhibited them to determine the effect this had on proliferation, migration, invasion, survival and the phosphoproteome.

**Methods:**

NF1-associated (ST88-14, 90-8, NMS2, NMS-PC, S462, T265-2c) and sporadic (STS-26T, YST-1) MPNST lines were used. Cells were transfected with doxycycline-inducible vectors expressing either a pan-inhibitor of the R-Ras subfamily [dominant negative (DN) R-Ras] or enhanced green fluorescent protein (eGFP). Methodologies used included immunoblotting, immunocytochemistry, PCR, Transwell migration, ^3^H-thymidine incorporation, calcein cleavage assays and shRNA knockdowns. Proteins in cells with or without DN R-Ras expression were differentially labeled with SILAC and mass spectrometry was used to identify phosphoproteins and determine their relative quantities in the presence and absence of DN R-Ras. Validation of R-Ras and R-Ras2 action and R-Ras regulated networks was performed using genetic and/or pharmacologic approaches.

**Results:**

R-Ras2 was uniformly expressed in MPNST cells, with R-Ras present in a major subset. Both proteins were activated in neurofibromin-null MPNST cells. Consistent with classical Ras inhibition, DN R-Ras and R-Ras2 knockdown inhibited proliferation. However, DN R-Ras inhibition impaired migration and invasion but not survival. Mass spectrometry-based phosphoproteomics identified thirteen protein networks distinctly regulated by DN R-Ras, including multiple networks regulating cellular movement and morphology. ROCK1 was a prominent mediator in these networks. DN R-Ras expression and *RRAS* and *RRAS2* knockdown inhibited migration and ROCK1 phosphorylation; ROCK1 inhibition similarly impaired migration and invasion, altered cellular morphology and triggered the accumulation of large intracellular vesicles.

**Conclusions:**

R-Ras proteins function distinctly from classic Ras proteins by regulating distinct signaling pathways that promote MPNST tumorigenesis by mediating migration and invasion.

**Plain English Summary:**

Mutations of the *NF1* gene potentially results in the activation of multiple Ras proteins, which are key regulators of many biologic effects. The protein encoded by the *NF1* gene, neurofibromin, acts as an inhibitor of both classic Ras and R-Ras proteins; loss of neurofibromin could cause these Ras proteins to become persistently active, leading to the development of cancer. We have previously shown that three related Ras proteins (the classic Ras proteins) are highly activated in malignant peripheral nerve sheath tumor (MPNST) cells with neurofibromin loss and that they drive cancer cell proliferation and survival by activating multiple cellular signaling pathways. Here, we examined the expression, activation and action of R-Ras proteins in MPNST cells that have lost neurofibromin. Both R-Ras and R-Ras2 are expressed in MPNST cells and activated. Inhibition of R-Ras action inhibited proliferation, migration and invasion but not survival. We examined the activation of cytoplasmic signaling pathways in the presence and absence of R-Ras signaling and found that R-Ras proteins regulated 13 signaling pathways distinct from those regulated by classic Ras proteins. Closer study of an R-Ras regulated pathway containing the signaling protein ROCK1 showed that inhibition of either R-Ras, R-Ras2 or ROCK1 similarly impaired cellular migration and invasion and altered cellular morphology. Inhibition of R-Ras/R-Ras2 and ROCK1 signaling also triggered the accumulation of abnormal intracellular vesicles, indicating that these signaling molecules regulate the movement of proteins and other molecules in the cellular interior.

**Video Abstract**

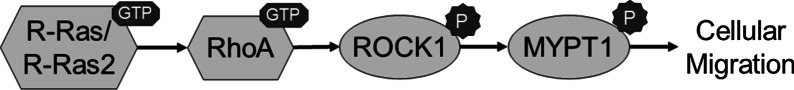

**Supplementary Information:**

The online version contains supplementary material available at 10.1186/s12964-021-00773-4.

## Background

The R-Ras subfamily of GTP-binding proteins (G-proteins) includes two proto-oncogenes—the closely related proteins R-Ras and R-Ras2/TC21. R-Ras and R-Ras2 are nearly ubiquitously expressed in human tissues, albeit at different levels [1, 2] (Human Protein Atlas; http://www.proteinatlas.org). In wild-type cells, Ras activation promotes proliferation, survival, migration, differentiation and/or other effects, depending upon the cellular context [3] and which Ras proteins are activated. Ras activation normally occurs when guanine nucleotide exchange factors (GEFs) catalyze the release of GDP from inactive Ras proteins and promote GTP binding which activates Ras; GEFs are themselves activated by upstream stimuli such as growth factor signaling [4–6]. The duration of Ras activation is limited by GTPase activating proteins (GAPs), which stimulate an intrinsic GTPase activity within Ras that converts GTP to GDP, thereby inactivating Ras [7, 8]. In human cancers, activating mutations in R-Ras subfamily genes bypass GAP-mediated regulation by impeding GTP hydrolysis, an event that leads to cellular transformation and tumorigenesis. In keeping with this, activating mutations of R-Ras2 occur in some human neoplasms in vivo and constitutively active R-Ras subfamily mutants induce transformation in vitro [9–20].

Ras proteins can also be aberrantly activated when cells lose expression of neurofibromin, a ubiquitously expressed Ras GAP that is encoded by the *neurofibromin 1* (*NF1)* gene. Neurofibromin loss has been linked to the pathogenesis of sporadically occurring CNS (glioblastomas [21–23]) and non-CNS (ovarian adenocarcinomas [24], adult acute myelogenous leukemia [25]) neoplasms. Most commonly, the pathogenesis of these neoplasms is driven by inactivating mutations of the *NF1* gene. However, neurofibromin expression can also be lost as a result of inappropriately increased proteolytic degradation [22, 26]. Since neurofibromin inactivates classic Ras proteins (H-, N- and K-Ras), M-Ras and R-Ras subfamily members [27], neurofibromin loss via either mechanism can potentially activate all six of these Ras proteins. However, it is currently unclear whether this actually occurs in neurofibromin-deficient neoplasms and, if so, what contribution each activated Ras protein makes to the pathogenesis of these tumors.

Germline inactivating mutations of *NF1* also occur in individuals with the familial tumor susceptibility syndrome Neurofibromatosis Type 1 (NF1). NF1 patients develop several types of tumors including benign tumors of peripheral nerve (neurofibromas) and aggressive malignancies derived from neurofibromas that are known as malignant peripheral nerve sheath tumors (MPNSTs). We and others have shown that *NF1*-null MPNSTs express all three classic Ras proteins, that these proteins are constitutively activated and that they promote proliferation and survival [27–33]. However, it is not known whether R-Ras subfamily members and M-Ras contribute to MPNST pathogenesis and, if so, what effects they exert. R-Ras subfamily members and M-Ras can be regulated by distinct GEFs and so are potentially activated by upstream signals different from those controlling classic Ras proteins [19, 27]. Further, little is known about the signaling pathways regulated by hyperactivated R-Ras subfamily members [10, 20, 34–36]. Much of the information that is available about these pathways has been obtained by over-expressing constitutively active mutants in tumor cells in vitro, which can lead to non-physiologic effector activation [37, 38] and misleading phenotypic effects [39].

We hypothesized that hyperactivated R-Ras subfamily members promote MPNST pathogenesis by eliciting physiologic effects distinct from those promoted by classic Ras proteins [33]. We first asked whether R-Ras, R-Ras2 and M-Ras are expressed in MPNST cells and if they are activated. Since R-Ras subfamily members, like classic Ras proteins [33], can potentially compensate for one another in MPNSTs, we used a dominant negative R-Ras mutant that globally inhibits R-Ras subfamily activation [40–42] to assess the effects of these proteins on MPNST proliferation, migration, invasion and survival and confirmed these effects using shRNAs targeting R-Ras or R-Ras2. We then used mass spectrometry-based phosphoproteome analyses to identify key signaling pathways potentially mediating the distinct effects of R-Ras proteins.

## Methods

### Antibodies, reagents and cell lines

See Additional file 8: Table S1 for a complete list of antibodies and reagents. We have previously described the sources of the human MPNST cell lines used in this study [41, 43]. These cell lines were regularly tested for *Mycoplasma* infection and their morphology and doubling times were regularly evaluated. Karyotypic analyses showed that these lines contained only human chromosomes, ruling out the possibility that they were contaminated with non-human cells.

### Immunoblotting

Cell lysates were resolved by polyacrylamide gel electrophoresis (PAGE), transferred to nitrocellulose and immunoblotted per our previously described methodology [42]. Blots were reprobed with an anti-GAPDH antibody to verify equal loading of lanes. SuperSignal Pico Chemiluminescence kits (Thermo Scientific) or an Odyssey Li-Cor Imaging System were used to detect immunoreactive species. Band intensities were quantified with ImageJ, using both the peak and ROI plugins.

### Conventional and real-time quantitative PCR

Conventional reverse transcription-polymerase chain reaction (RT-PCR) was performed as previously described [42]. Real-time quantitative PCR was performed using TaqMan primers with an Applied Biosystems 7500 Real-Time PCR System (Applied Biosystems) as previously described [43]. Data generated in these experiments were analyzed with Applied Biosystems Sequence Detection software (version 1.4). The RT-PCR and real-time qPCR primers used in these experiments are described in Additional file 9: Table S2 and Additional file 10: Table S3, respectively.

### Ras dominant negative mutants and epitope-tagged Ras expression plasmids

A plasmid expressing an HA-epitope tagged R-Ras dominant negative (DN) mutant was acquired from the Missouri S&T cDNA Resource Center (Rolla, MO; Additional file 11: Table S4). The plasmid insert was cloned into the *Kpn*I and *Eco*RV sites of pBIG2i [44] to produce a doxycycline-inducible DN R-Ras plasmid (pSLC658). The construction of the pBIG2i-based doxycycline-inducible GFP control (pSLC460) and DN H-Ras (pSLC703) plasmids and the method used to generate MPNST cell lines stably transfected with these plasmids has been previously described [33].

Plasmids expressing untagged wild-type R-Ras and R-Ras2/TC21 were also obtained from the Missouri S&T cDNA Resource Center (Additional file 11: Table S4). Three single-nucleotide deletions in the R-Ras2 plasmid were corrected with a QuikChange II Site-Directed Mutagenesis kit (Stratagene). Wild-type sequences were PCR amplified using primers containing *Not*I or *Xho*I sites, digested with *Not*I and *Xho*I and cloned into *Not*I/*Xho*I digested Myc-tagged pcDNA3.1 + to create Myc-tagged R-Ras (pSLC891) and R-Ras2 (pSLC901) expression vectors.

### Transfection of MPNST cell lines

Transient transfections were performed using Fugene 6 Transfection Reagent (Roche) in serum-free DMEM as recommended by the manufacturer. For stable transfection of the doxycycline-inducible DN R-Ras plasmid, transfected cells were split into tetracycline-free DMEM-10 and selected with hygromycin (50–100 µg/ml, empirically determined for each line). Clones were picked two weeks later and screened by immunoblotting to identify those with HA-epitope tag expression when cultured in media containing 2 µg/ml doxycycline, but not when maintained in doxycycline-free media. Stable transfectants were maintained in tetracycline-free DMEM-10 (DMEM, 10% tetracycline-free FBS, 10 U/ml penicillin, 10 µg/ml streptomycin) supplemented with hygromycin at the concentration used for initial selection.

### Ras activation assays

Human MPNST cells were transiently transfected with plasmids expressing Myc-tagged wild-type R-Ras, Myc-tagged wild-type R-Ras2, HA-tagged DN R-Ras, HA-tagged DN H-Ras, or eGFP. Cells were grown in DMEM-10 and lysed at 70–90% confluency in magnesium-containing lysis buffer [25 mM HEPES (pH 7.5), 150 mM NaCl, 10 mM MgCl_2_, 1 mM EDTA, 1% Igepal CA-630, 10% glycerol] supplemented with protease (Sigma #P8340, 1:100 dilution) and HALT phosphatase (Thermo Scientific #78420, 1:100 dilution) inhibitor cocktails. Lysates were clarified by centrifugation (20,000x*g* for 10 min) and the supernatant retained for analysis. Protein concentrations were determined using a DC Assay kit (Bio-Rad) per the manufacturer’s recommendations. 250 µg of each lysate and 20 µl of Ras Assay Reagent (Raf-1 Ras-binding domain agarose beads; Millipore, Lake Billerica, MA) were mixed to capture activated Ras per the manufacturer’s instructions. Beads were washed to remove unbound proteins and then boiled for 15 min in 40 µl 2× Stop Buffer [250 mM Tris–HCl (pH 6.8), 5 mM EDTA, 5 mM EGTA, 2% SDS, 10% glycerol, 25 mM dithiothreitol, 300 µM bromophenol blue] to release bound activated Ras. Captured activated Ras and corresponding whole cell lysates were resolved on 12% SDS-PAGE gels, immunoblotted, and probed as described above.

### RhoA activation assays

Human MPNST lines stably transfected with doxycycline-inducible DN R-Ras were grown in DMEM-10. Lysates were collected and prepared under the conditions stated above for the Ras activation assay. 500 µg of each lysate and 12 µl of RhoA Assay Reagent (Rhotekin Rho-binding domain agarose beads; Millipore, Lake Billerica, MA) were mixed to capture activated RhoA per the manufacturer’s instructions. Beads were washed to remove unbound proteins and then boiled for 15 min in 40 µl 2× Stop Buffer [250 mM Tris–HCl (pH 6.8), 5 mM EDTA, 5 mM EGTA, 2% SDS, 10% glycerol, 25 mM dithiothreitol, 300 µM bromophenol blue] to release bound activated RhoA. Captured activated RhoA and corresponding whole cell lysates were resolved on 4–15% gradient SDS-PAGE gels, immunoblotted, and probed as described above.

### ^3^H-Thymidine proliferation assays

Cells stably transfected with doxycycline-inducible vectors encoding DN R-Ras or eGFP were plated in tetracycline-free DMEM-10 in the presence or absence of 2 µg/ml doxycycline (5,000 cells/well in 48-well plates). 48 h later, ^3^H-thymidine incorporation assays were performed as previously described [43]. Sixteen replicates were counted per condition.

### Cell viability assays

MPNST cells were plated at 20,000 cells per well in DMEM-10 overnight and then cultured in either DMEM-10 or serum-free DMEM for an additional 24 h in the presence or absence of 2 μg/ml doxycycline. After two rinses with Hanks’ Balanced Salt Solution (HBSS), cells were incubated for 30 min at room temperature in 200 μl of 4 ng/ml calcein acetoxymethyl (AM) diluted in HBSS. Signals were measured with a fluorescent plate reader.

#### Transwell migration assays

Cell lines stably transfected with doxycycline-inducible vectors encoding DN R-Ras or eGFP were maintained for 48 h in tetracycline-free DMEM-10 supplemented with vehicle or 2 µg/ml doxycycline. Cells were then cultured in Schwann cell-defined medium [45, 46] supplemented with vehicle or 2 µg/ml doxycycline. After 24 h, migration assays were performed on poly-L-lysine/laminin coated Transwell filter inserts (Becton Dickinson Labware) as previously described [47]. For experiments using ROCK inhibitor Y27632 (Selleck Chemicals), inhibitor was added 30 min after plating to allow cell adherence prior to beginning the 6-h migration period. Cells that migrated through the filters were stained with 10 µg/ml Hoescht and photographed using fluorescence microscopy at 10× magnification with an EVOS M5000 Imaging System (Thermo Scientific). Cell counts from 9 distinct fields per filter were quantified using ImageJ [48].

#### 3D invasion assay

Lines stably transfected with doxycycline-inducible DN R-Ras or eGFP vectors were maintained for 48 h in tetracycline-free DMEM-10 supplemented with vehicle or 2 µg/ml doxycycline. Cells were then plated in 8-well chamber slides (Corning) as a single cell suspension of 5000 cells per well on a 43.5 µl/cm^2^ base layer of growth factor reduced Matrigel (Corning) in growth media supplemented with 2% Matrigel and vehicle or 2 µg/ml doxycycline. Cells were allowed to grow for 4 days before imaging with an EVOS M5000 Imaging System (Thermo Scientific). Subsequent immunostaining and imaging was performed as previously described [49].

#### Immunocytochemistry

Matrigel-coated monolayer and 3D Matrigel cultures plated in 8-well chamber slides were washed in PBS and then fixed for 15 min in 2% paraformaldehyde in PBS. Slides were washed 3× with PBS (5 min/wash) and then permeabilized for 10 min with 0.5% Triton-X100. Slides were rewashed for 5 min in PBS and blocked (10% goat serum, 5% BSA, and 0.5% Tween-20 in PBS) for 1 h at room temperature. Slides were washed 4× (5 min/wash) before a 1-h room temperature incubation with Phalloidin CF568 conjugate counterstain (Biotium) diluted 1:500 in blocking buffer. Slides were washed 4× (5 min/wash) in PBS and mounted in Prolong Diamond Antifade mounting media with DAPI (Life Technologies).

For non-Matrigel monolayer cultures, MPNST cells were plated on poly-L-lysine/laminin coated #1.5 coverslips (Electron Microscopy Sciences) and grown to 60–80% confluency before fixing and blocking as described above. Coverslips were then incubated overnight at 4 °C with primary antibodies, followed by washing and incubation with isotype matched secondary antibody and Phalloidin ActinGreen™ 488 ReadyProbes™ Reagent (Thermo Fisher). Coverslips were removed from culture wells and mounted on Fisherbrand Superfrost Plus microscope slides (Fisher Scientific) with Prolong Glass Antifade mounting media with DAPI (Life Technologies). Slides were imaged at 20× and 63× on an inverted microscope (Zeiss) and images analyzed using ImageJ [48].

#### Phosphoproteome analyses

Invitrogen’s SILAC (stable-isotopic labeling using amino acids in culture) Protein and Quantitation Media Kit with DMEM-flex (MS10030) was used per the manufacturers’ recommendations to label MPNST cells stably transfected with doxycycline-inducible DN R-Ras with either light (^12^C_6_) or heavy (^13^C_6_) L-lysine as previously described [33]. Phosphoproteins were isolated from cell lysates using a Pierce Phosphoprotein Enrichment Kit (90,003). Isolated phosphoproteins were concentrated with an ICON concentrator and resolved using SDS-PAGE. Protein bands were stained with GelCode Blue Stain Reagent (Thermo Scientific) and gel lanes divided into slices of equal size. After destaining overnight with 1:1 100 mM ammonium bicarbonate:acetonitrile, gel slices were incubated with 25 mM dithiothreitol at 50 °C for 30 min to reduce disulfide bonds and then incubated for 30 min in the dark with 55 mM iodoacetamide to alkylate free thiol groups. Following two washes with 100 mM ammonium bicarbonate (30 min/wash), gel slices were dried in a SpeedVac (Savant) and then digested overnight at 37 °C with 12.5 ng/µl Promega Gold Mass Spectrometry Grade Trypsin. Peptides were extracted from the digested gel slices with two 15-min incubations in a 1:1 mixture of 5% formic acid:50% aqueous acetonitrile. Extracted peptides were pooled, dried, and resuspended in 20 µl of 0.1% formic acid.

An Eksigent autosampler was used to load 2 µl of each digest (2 µl/min) on a 2 cm × 75 µm i.d. PepMap100 C_18_ reverse-phase trap cartridge (Dionex; Sunnyvale, CA). Following a 4-min wash with 0.1% formic acid in ddH_2_0, bound peptides were flushed onto a 15 cm × 75 µm i.d. PepMap100 C_18_ reverse-phase analytical column (Dionex) with a 40-min linear (5–50%) acetonitrile gradient in 0.1% formic acid (300 nl/min) using an Eksigent Nano1D + LC. The column was washed for 15 min with 90% acetonitrile-0.1% formic acid and then re-equilibrated for 30 min with 5% acetonitrile/0.1% formic acid. An Applied Biosystems 5600 Triple-ToF mass spectrometer (AB-Sciex) was used to analyze the protein digest (IonSpray voltage, 2300 V; declustering potential, 60 V). Ionspray and curtain gases were at 10 psi and 20 psi, respectively, and the interface heater temperature was 120 °C. A time-of-flight survey scan from 400 to 1250 *m/z* was performed to identify the top twenty most intense ions for MS/MS analysis. Product ion time-of-flight scans at 50 ms were performed to obtain the tandem mass spectra of the selected parent ions over the *m/z* 400–2000 range. Analyst software (AB Sciex; version TF) was used to centroid and de-isotope the spectra. The mass accuracy of the mass spectrometer was confirmed using a β-galactosidase trypsin digest. ProteinPilot software (version 4.0; AB Sciex) was used to assess the relative abundance of heavy versus light-labeled proteins from tryptic peptide spectra via the Paragon algorithm. After removing contaminating proteins, reversed proteins, and proteins with only one quantifiable peptide, results were manually bias corrected in ProteinPilot (AB Sciex), using the median H:L ratio of all proteins in the data set for a given experiment as a correction factor. Reported proteins were identified with ≥ 99% confidence by ProteinPilot (unused score ≥ 2), with a false discovery rate < 1%. Network analyses were performed using only those proteins in which the |log_2_ (heavy-to-light ratio)|> 0.33 with Ingenuity Pathway Analysis software (Ingenuity Systems). Fisher’s exact test was used to determine the probability that the changes in the networked proteins could occur by random chance; the –log of this *p*-value is reported as the IPA score.

#### shRNA-mediated gene expression knockdowns

Sigma shRNA lentiviral vectors targeting R-Ras (TRCN0000047883, TRCN0000047885, TRCN0000047887) or R-Ras2 (TRCN0000047723, TRCN0000047724, TRCN0000047727) were obtained from the MUSC Hollings Cancer Center shRNA Technology Shared Resource. Virus was generated by infecting HEK 293T cells with the shRNA vectors and MISSION shRNA Lentiviral Packaging Mix (Sigma product #SHP001), according to the MISSION shRNA user manual. Transfections were performed in the presence of 10% antibiotic-free DMEM. Viral supernatant was harvested 24 h after transfection and 1 ml aliquots were prepared and stored at − 80° for future use.

MPNST cells were infected with lentiviruses following a reverse transduction protocol. Cells were harvested and seeded into 60 cm dishes (300,000 cells/dish) with 0.5 ml of viral supernatant, 1.5 ml of 10% DMEM, and polybrene at a final concentration of 10 mg/ml. Selection was achieved by the addition of puromycin 48 h following transduction. Cells were selected for at least 72-h after the addition of puromycin before validating knockdown by real time-PCR.

#### Statistical analysis

All experiments were performed and quantified using at least 3 independent biological and technical replicates, with individual replicates indicated in assay descriptions. Data were normalized to uninduced control data and graphs were plotted with normalized mean ± SEM. P-values were calculated using a two-tailed Student *t*-test, one-way ANOVA or two-way ANOVA as indicated using GraphPad Prism version 8.0.0 for Windows (GraphPad Software, San Diego, California USA).

## Results

### R-Ras and R-Ras2 are expressed and activated in human MPNST cells

**Fig. 1:**
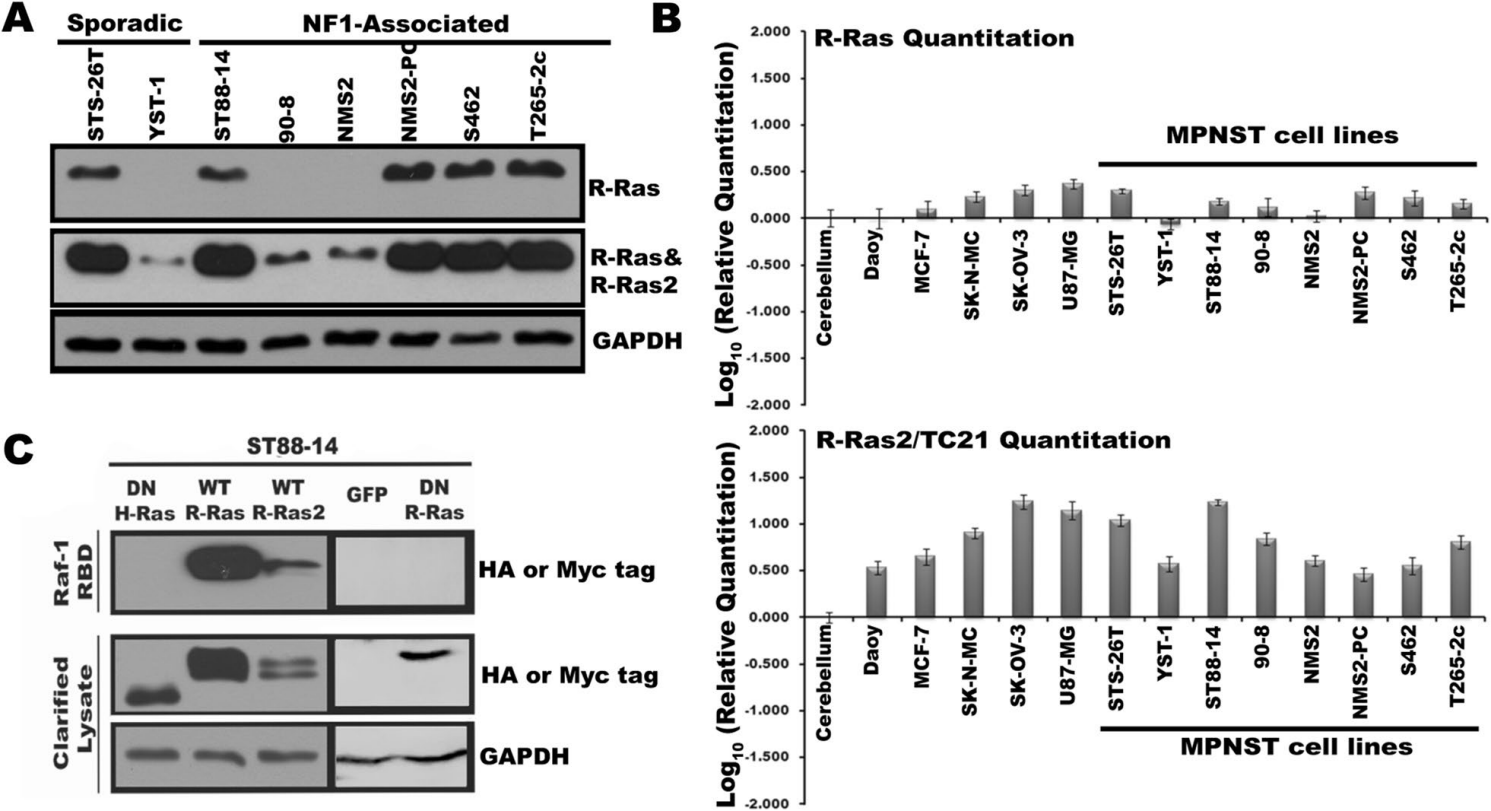
Human MPNST cell lines variably express R-Ras subfamily members. **a** Lysates from eight human MPNST cell lines probed with antibodies recognizing R-Ras or both R-Ras and R-Ras2 proteins. Blots were probed with an anti-GAPDH antibody to confirm equal loading. Antibody dilutions were: R-Ras 1:10,000, R-Ras plus R-Ras2 (Abnova H000022800_M01) 1:10,000 and GAPDH 1:100,000. **b** R-Ras and R-Ras2 transcript levels in MPNST lines were quantitated using real-time PCR and compared to the level of expression evident for each transcript in normal human cerebellum tissue, Daoy medulloblastoma cells, MCF-7 breast adenocarcinoma cells, SK-N-MC neuroepithelioma cells, SK-OV-3 ovarian adenocarcinoma cells and U87-MG glioblastoma cells. **c** Raf-1 RBD pulldown assays performed on ST88-14 cells transiently transfected with plasmids directing the expression of Myc-tagged wild-type R-Ras, Myc-tagged wild-type R-Ras2, HA-epitope tagged dominant negative H-Ras plasmid, HA-epitope tagged dominant negative R-Ras, or eGFP. Only activation of wild-type R-Ras and R-Ras2 is evident in these cells. Upper panel is the material captured with Raf-1 RBD probed with antibodies recognizing the HA or Myc epitope tags on the recombinant proteins. The bottom two panels are clarified lysates probed for the epitope tags or GAPDH, respectively

We examined R-Ras and R-Ras2 expression in a panel of eight MPNST lines that had different *NF1* mutations and levels of neurofibromin expression. Six of these lines (ST88-14, 90-8, NMS2, NMS2-PC, S462 and T265-2c cells) were derived from NF1-associated MPNSTs, while the other two lines (STS-26T and YST-1 cells) were from sporadic MPNSTs; their levels of neurofibromin protein expression and *NF1* mutational status are indicated in Additional file 2: Fig. S1A and B. Given the high homology of R-Ras and R-Ras2, we tested the specificity of a panel of anti R-Ras and R-Ras2 antibodies against immunoprecipitates isolated from ST88-14 MPNST cells transiently transfected with Myc-tagged wild-type R-Ras, R-Ras2, H-Ras, N-Ras or K-Ras expression plasmids; the latter three plasmids were included to verify that tested antibodies did not cross-react with classic Ras proteins. Exogenously expressed Ras proteins were immunoprecipitated from cell lysates using an anti-Myc epitope tag antibody. The resulting immunoprecipitates were then probed with candidate isoform-specific Ras antibodies. This approach identified an anti R-Ras antibody that recognized its target antigen and did not cross-react with R-Ras2 (Additional file 3: Fig. S2, top panel). Although none of the six anti-R-Ras2 antibodies tested were R-Ras2 specific, we did identify four antibodies that recognized both R-Ras and R-Ras2 and did not cross-react with H-Ras (Additional file 3: Fig. S2), N-Ras or K-Ras (not shown). We used the R-Ras antibody and the R-Ras/R-Ras2 antibody from Abnova (top two panels, Additional file 3: Fig. S2) for all subsequent experiments. We found that R-Ras expression was variable, being detectable in five of the eight lines (Fig. 1a). Of note, R-Ras protein was evident in NMS2-PC cells, but not NMS2 cells despite the fact that the NMS2-PC line was derived from a metastasis of the primary MPNST that was the source of the NMS2 line. The antibody recognizing both R-Ras and R-Ras2 detected proteins of the expected molecular mass in all eight lines, with more intense bands evident in lines that also had R-Ras expression (Fig. 1a).

To complement our protein data, we examined the expression of R-Ras and R-Ras2 transcripts in our panel of MPNST cell lines using real-time quantitative PCR. R-Ras transcripts were evident in the same five MPNST lines with detectable R-Ras protein (Fig. 1b, top) and were present at levels similar to those seen in some cell lines derived from other cancer types (SK-N-MC neuroepithelioma, SK-OV-3 ovarian cancer and U87-MG glioblastoma cells). Lower levels of R-Ras mRNA were evident in the same three MPNST lines (YST-1, 90-8, NMS2) that did not have detectable R-Ras protein. Although there was some variability in the levels of R-Ras2 mRNA detected, all eight lines showed expression of R-Ras2 that was greater than that seen in normal human cerebellum and comparable to that in several other non-MPNST cancer cell lines (Fig. 1b, bottom). These findings indicate that only R-Ras2 expression was ubiquitously expressed in our MPNST cell line panel.

We also examined expression of mRNA encoding M-Ras in this panel of human MPNST cell lines and found that M-Ras transcripts were detectable in only one of the eight lines we examined (Additional file 2: Fig. S1C). Further, we could not detect M-Ras protein in any of these eight cell lines by immunoblotting with an anti M-Ras antibody (data not shown). M-Ras is more distantly related to R-Ras and R-Ras2 (Additional file 2: Fig. S1D) and often has actions more similar to those of classic Ras proteins [50]. Given this and the lack of M-Ras expression in MPNST cells, we focused on R-Ras and R-Ras2 in all of our subsequent experiments.

In preliminary experiments examining the activation of endogenous R-Ras and R-Ras2 in MPNST cells, we found that, despite their specificity, our antibodies did not effectively detect activated Ras proteins in Raf1 pulldown assays. Consequently, we examined the activation of these proteins in lysates of ST88-14 cells transiently transfected with plasmids directing the expression of Myc epitope-tagged wild-type R-Ras or R-Ras2. We then compared the level of Ras activation in these cells to that in cells transiently transfected with vectors expressing DN R-Ras, DN H-Ras or enhanced green fluorescent protein (eGFP). The Raf-1 Ras binding domain (RBD) affinity reagent, which only binds activated Ras, pulled down Myc-tagged wild-type R-Ras and R-Ras2 (Fig. 1c). In contrast, the HA-tagged DN R-Ras and H-Ras mutants—which exist in an inactive GDP-bound conformation due to their altered nucleotide-dissociation kinetics [51]—did not bind to the Raf1 Ras-binding domain; binding was also not evident in cells with induced expression of eGFP. These observations indicate that R-Ras and R-Ras2 are activated in ST88-14 cells.

### Dominant negative R-Ras inhibits the proliferation, migration, and invasion of human MPNST cells, but not their survival

**Fig. 2:**
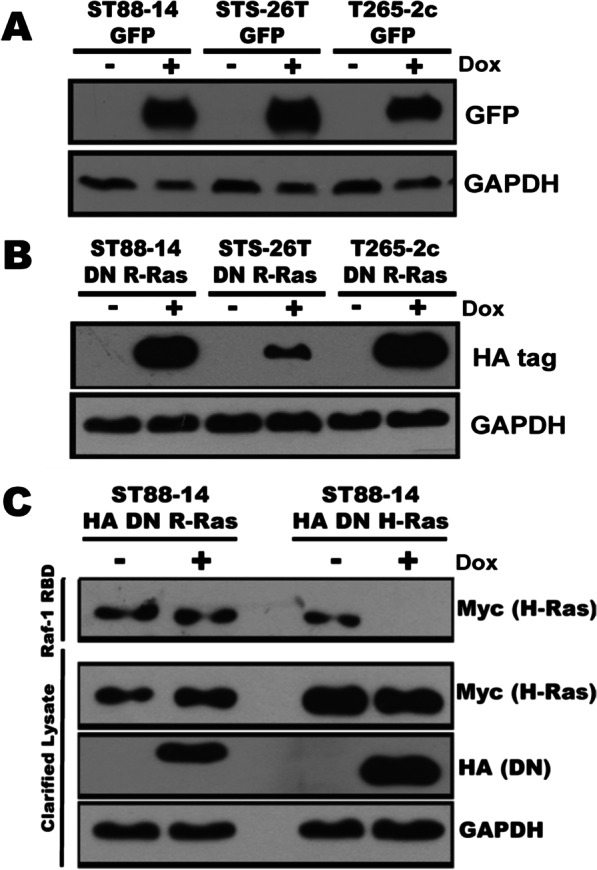
Assessment of the regulation of doxycycline-inducible plasmids expressing eGFP or DN R-Ras stably tranfected into MPNST cells. **a**, **b** Expression of eGFP (**a**) or HA-tagged DN R-Ras (**b**) in stably transfected ST88-14, STS-26T and T265-2c cells 72 h after the cells were challenged with 2 μg/ml doxycycline. **c** Raf-1 RBD pulldown assays in ST88-14 MPNST cells stably transfected with doxycycline-inducible vectors expressing DN R-Ras or DN H-Ras and transiently transfected with a plasmid expressing Myc-tagged H-Ras. DN H-Ras, but not DN R-Ras, inhibits H-Ras activation

Our finding that R-Ras and R-Ras2 are activated in MPNST cells led us to ask what function(s) these proteins perform. To answer this question, we stably transfected doxycycline-inducible vectors expressing HA-epitope tagged DN R-Ras, which inhibits the activation of both R-Ras and R-Ras2 [40], or eGFP into ST88-14, STS-26T and T265-2c MPNST cells. Expression of GFP or DN R-Ras in these cells was undetectable in the absence of doxycycline and maximally induced by 1.0–2.0 μg/ml doxycycline (Fig. 2a, b); we have previously shown that these doxycycline concentrations have no effects on MPNST cell DNA synthesis or migration [33]. Upon examining the expression of the DN mutant at different times post-doxycycline exposure, we found that maximal expression was achieved 48 h after the addition of 2 µg/ml doxycycline in ST88-14 cells (Additional file 2: Fig. S1E, F). These same conditions similarly induced the expression of eGFP and DN R-Ras in all three cell lines (data not shown) and so were adopted for subsequent experiments.

Some GEFs are utilized by both R-Ras and classic Ras proteins, which raises the concern that the DN R-Ras mutant might also effect classic Ras protein signaling in MPNST cells. To examine this, we transiently transfected a plasmid expressing Myc-tagged H-Ras into ST88-14 cells stably transfected with doxycycline-inducible vectors expressing either DN R-Ras or DN H-Ras. We then performed Raf RBD pulldown assays in the presence or absence of DN mutant expression. We found that our previously described DN H-Ras mutant [33] effectively inhibited the activation of Myc-tagged H-Ras in ST88-14 cells (Fig. 2c). In contrast, the expression of the DN R-Ras mutant had no effect on the activation of H-Ras.

**Fig. 3:**
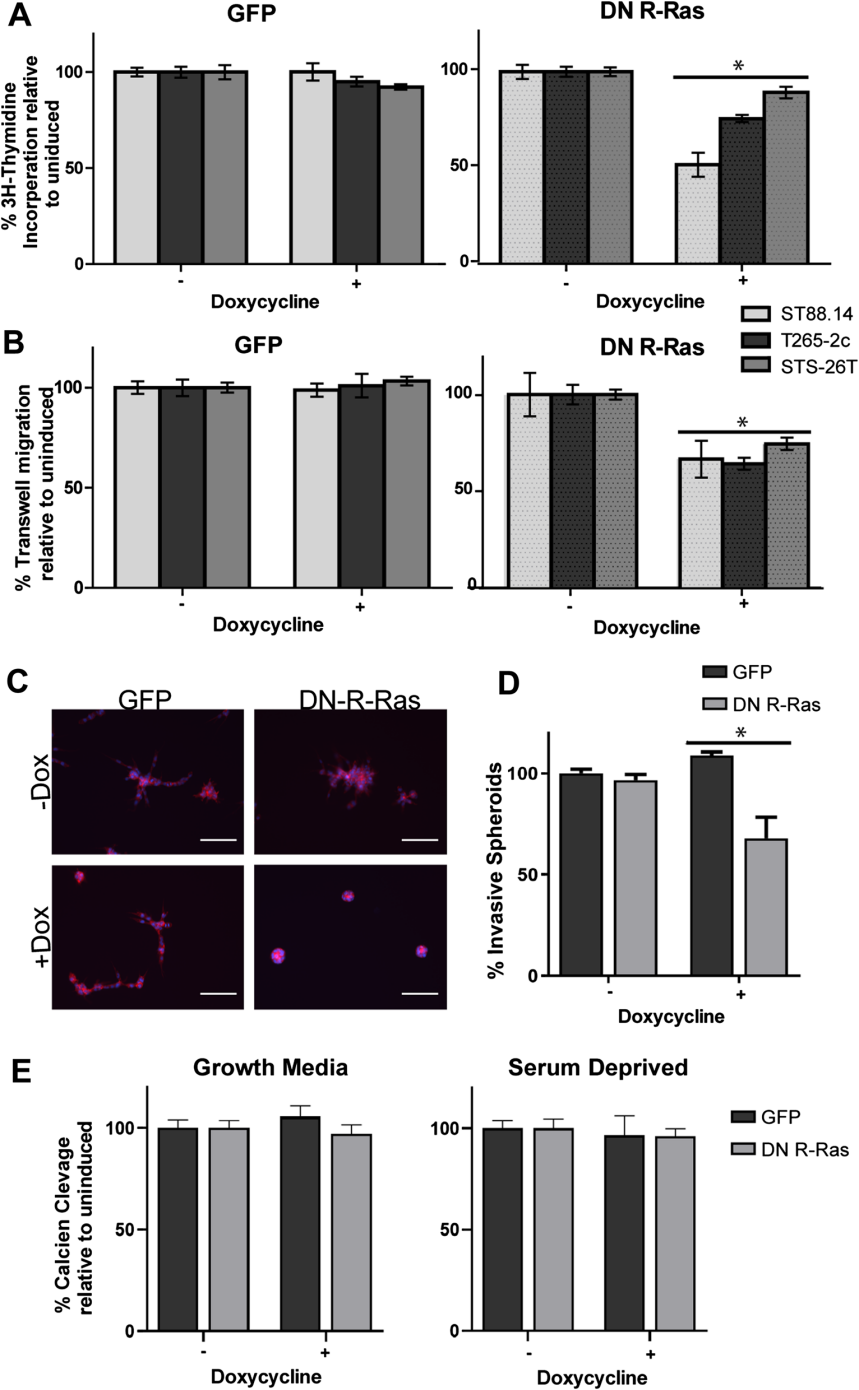
Induction of DN R-Ras expression inhibits MPNST proliferation, migration and invasion but not survival. **a** Induction of DN R-Ras expression, but not expression of eGFP, reduces ^3^H-thymidine incorporation in the ST88-14, STS-26T, and T265-2c doxycycline-inducible stable cell lines relative to uninduced controls. 16 replicates were performed for each condition. **b** Induction of DN R-Ras expression, but not eGFP expression, reduces Transwell migration in ST88-14, STS-26T, and T265-2c cells. **c** Immunofluorescence microscopy of phalloidin-stained ST88-14 MPNST cells grown in 3D Matrigel-matrix cultures. Left images are of ST88-14 cells stably transfected with a doxycycline-inducible eGFP expression vector and right images are the same cell line stably transfected with a doxycycline-inducible DN R-Ras vector. Upper panels are uninduced cells and lower panels are cells with expression induced by doxycycline. Scalebar = 400 µm (**d**) Quantification of percentage of spheroids in 3D culture with invasive phenotype, defined by circularity < .65. **e** Calcein AM cleavage assays in ST88-14 cells grown in the absence (left) or presence (right) of serum. Induction of eGFP and DN R-Ras expression had no statistically significant effect on the number of viable cells. For all panels, * indicates *p*-value < .0001 for comparison to controls

To determine whether R-Ras and/or R-Ras2 signaling promotes the proliferation of MPNST cells, we performed ^3^H-thymidine incorporation assays in these cells in the presence and absence of DN R-Ras expression. We found that expression of DN R-Ras, but not the eGFP control, produced statistically significant reductions in ^3^H-thymidine incorporation in ST88-14, T265-2c and STS-26T cells compared to uninduced cells (Fig. 3a). The observed effects in different lines were not equivalent, however. ST88-14 cells had the greatest reduction in ^3^H-thymidine incorporation, while STS-26T cells had a smaller, but still statistically significant, reduction in ^3^H-thymidine incorporation. The effect of DN R-Ras on the proliferation of T265-2c cells was intermediate between the two. These findings argue that R-Ras proteins, like classic Ras proteins, promote the proliferation of MPNST cells [33].

We next assessed the effect that DN R-Ras exerted on MPNST migration using Transwell migration assays performed with experimental parameters that we previously optimized for MPNST cells [47]. We have previously shown that induction of the expression of DN H-Ras, a pan-inhibitor of classic Ras proteins, in these same three MPNST cell lines had no effect on their migration [33]. Conversely, induction of DN R-Ras expression resulted in a statistically significant inhibition of the migration of ST88-14 and T265-2c MPNST cells, while induction of the eGFP negative control had no effect (Fig. 3b). Interestingly, the sporadic STS-26T MPNST cell line, which expresses wild-type neurofibromin and does not demonstrate hyperactivation of classic Ras proteins [52], also exhibited a reduction in migration following DN R-Ras induction comparable to that seen in the ST88-14 and T265-2c lines. Thus, while both classic Ras and R-Ras subfamily proteins promote MPNST cell proliferation, only R-Ras proteins drive the migration of these MPNST cells.

To determine whether R-Ras subfamily members are required for MPNST cell invasion, we performed 3D cultures with ST88-14 cells stably transfected with doxycycline-inducible DN R-Ras or eGFP vectors. In these experiments, suspensions of cells that had been previously incubated for 48 h with vehicle or doxycycline were plated onto Matrigel and grown as 3D spheroid cultures for four more days. To demonstrate their morphology, the actin cytoskeleton of the cells was stained with CF568-conjugated phalloidin. In the absence of doxycycline induction (unimpaired R-Ras/R-Ras2 signaling), cells stably transfected with either DN R-Ras or eGFP vectors showed a highly invasive growth phenotype as determined by characteristic actin filament projections invading the matrix (Fig. 3c). However, when DN R-Ras expression was induced with doxycycline exposure, there was a notable reduction in invasion relative to the eGFP induced controls. Quantification of this invasion showed that induction of DN R-Ras produced a statistically significant reduction in the percentage of invasive spheroids (Fig. 3d). We also attempted to establish spheroid cultures with STS-26T and T265-2c cells stably transfected with the doxycycline-inducible DN R-Ras vector. However, although the cells were viable, we could not get these two lines to grow as spheroid cultures following DN R-Ras induction and thus were unable to assess the effect that DN R-Ras exerts on their invasion. Nonetheless, our observations with ST88-14 cells indicate that R-Ras and/or R-Ras2 promote the invasion of at least some MPNST cell lines.

We previously showed that expression of DN H-Ras impairs the survival of MPNST cells grown under stressed conditions (serum-free media); death under these conditions is due to caspase-dependent apoptosis [33]. To determine whether DN R-Ras similarly inhibited MPNST survival under the same conditions, we compared the viability of ST88-14 cells expressing DN R-Ras in serum-containing DMEM to the viability of the same cells in serum-free DMEM. In serum-containing media, ST88-14 cell viability, as assessed by calcein AM cleavage, was not affected by induction of either DN R-Ras or eGFP expression (Fig. 3e). When we performed this same experiment in ST88-14 cells grown in serum-free DMEM, we found that the number of viable cells present was not significantly reduced following the induction of either DN R-Ras or eGFP; in parallel experiments, induction of DN H-Ras expression reduced the number of viable cells. Consequently, in contrast to what is seen when classic Ras signaling is inhibited, inhibition of R-Ras signaling does not reduce MPNST survival under these conditions of non-glucose nutrient stress.

### Expression of DN R-Ras induces distinct changes in the phosphoproteome

Our observation that DN R-Ras and DN H-Ras similarly inhibited proliferation, while having differing effects on migration suggested that R-Ras proteins, unlike classic Ras proteins, regulate cytoplasmic signaling cascades that control migration. To identify proteins whose phosphorylation was regulated by R-Ras proteins, we examined the phosphoproteome of ST88-14 cells in the presence and absence of DN R-Ras expression. These experiments were performed using the same methodology we previously used to identify the effects that expression of doxycycline-inducible DN H-Ras had on the phosphoproteome of ST88-14 MPNST cells [33]. In this methodology, doxycycline-induced cells were labeled with media containing a heavy (^13^C_6_) lysine isotope, while uninduced cells were labeled with media containing a light (^12^C_6_) lysine isotope. Equal quantities of light- and heavy-labeled cell lysates were then mixed and fractionated on gallium (Ga^2+^) immobilized metal ion affinity chromatography (IMAC) columns, which bind proteins containing negatively charged phosphate groups. Immunoblotting for phosphorylated Erk1/2^Thr202/Tyr204^ as a representative phosphoprotein confirmed that these proteins were enriched in the proteins binding to the IMAC columns (Additional file 4: Fig. S3).

**Fig. 4:**
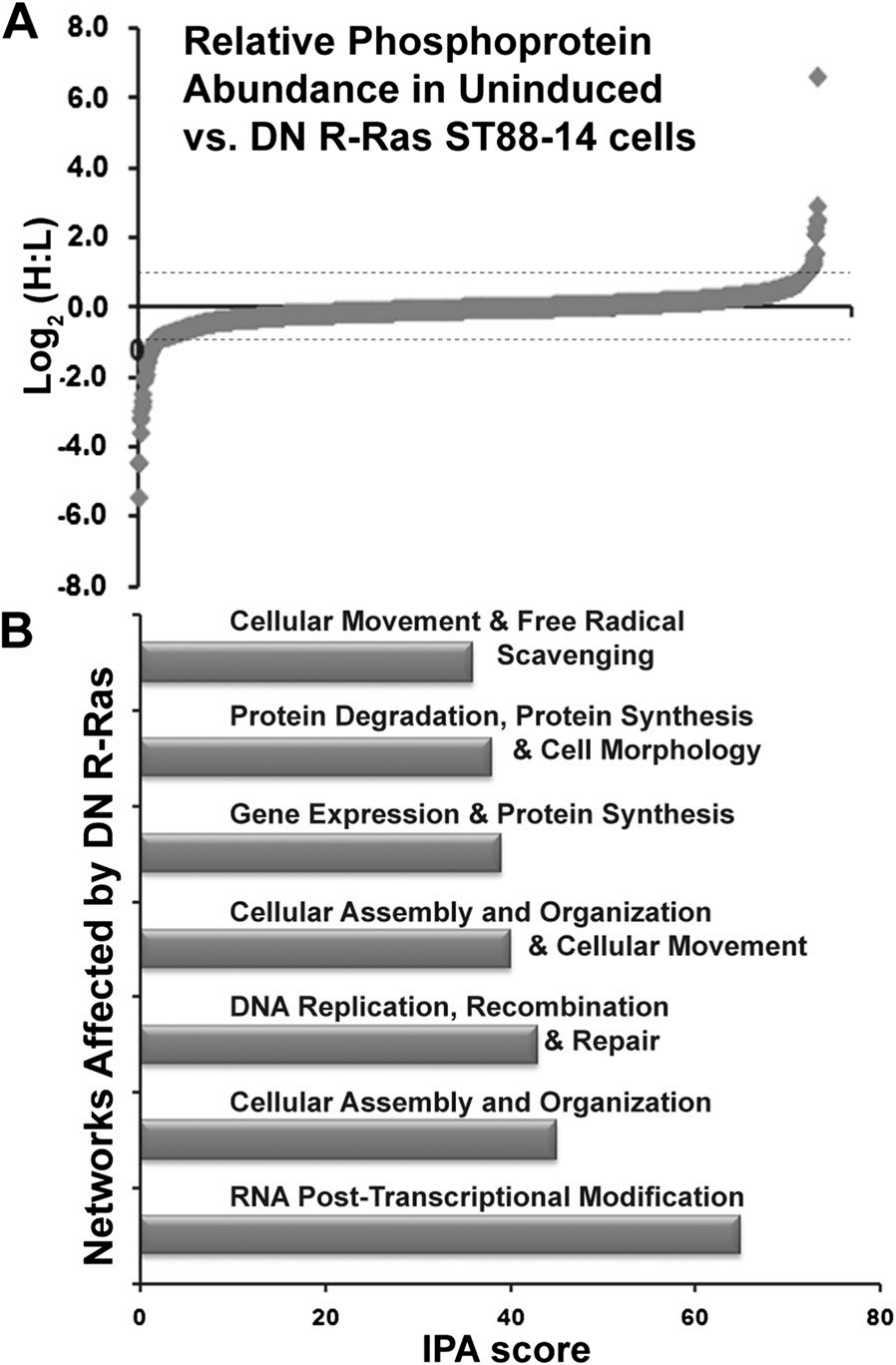
Distinct networks of phosphorylated proteins are affected by DN R-Ras expression. **a** The global distribution of heavy (DN R-Ras) to light (uninduced) ratios of detected phosphoproteins in ST88-14 MPNST cells. Plotted values are log_2_(H:L). Note that the phosphorylation of the vast majority of detected proteins are unaffected by DN R-Ras expression. **b** IPA analysis identified thirteen networks affected by DN R-Ras induction with IPA scores ≥ 16; the seven top ranked networks are shown. The proteins in the top seven networks whose phosphorylation is altered by DN R-Ras expression are presented in Table 2

Liquid chromatography-tandem mass spectrometry (LC–MS/MS) was then used to determine the identity and relative abundance of each light-labeled versus heavy-labeled protein captured by IMAC based on the relative intensities of heavy and light tryptic peptides (Additional file 5: Fig. S4). The phosphorylation status of most of the detected proteins did not change following induction of DN R-Ras (Fig. 4a), as indicated by the tight clustering of heavy-to-light (H:L) ratios around 1 (log_2_(H:L) = 0) in these populations of proteins. However, there was a subpopulation of proteins present that both had substantial differences in phosphorylation after induction of DN R-Ras expression (|log_2_(H:L)|> 0.33) and met criteria for statistically significant detection (i.e., multiple peptides from each protein were detected with statistically significant changes). We compared the set of proteins whose phosphorylation was altered following induction of DN R-Ras expression to the set of proteins we have previously shown to have altered phosphorylation following induction of DN H-Ras expression [33]. This comparison identified a set of proteins whose phosphorylation was altered by DN R-Ras expression but not by DN H-Ras. The most significant hits in the set of proteins whose phosphorylation was specifically altered by DN R-Ras are shown in Table 1.

**Table 1:** Proteins significantly altered by DN R-Ras

Accession #	Name	Manual bias corrected H:L	Protein pilot
			*p*-value
sp|P01857|IGHG1	Ig gamma-1 chain C region GN = IGHG1	0.0452	< 0.05
sp|Q99880|H2B1L	Histone H2B type 1-L GN = HIST1H2BL	0.1288	< 0.05
sp|P04114|APOB	Apolipoprotein B-100 GN = APOB	0.1598	< 0.05
sp|P38159|HNRPG	Heterogeneous nuclear ribonucleoprotein G GN = RBMX	0.1826	< 0.01
sp|P62979|RS27A	Ubiquitin-40S ribosomal protein S27a GN = RPS27A	0.4515	< 0.001
sp|Q13464|ROCK1	Rho-associated protein kinase 1 GN = ROCK1	0.4556	< 0.05
sp|P62987|RL40	Ubiquitin-60S ribosomal protein L40 GN = UBA52	0.6324	< 0.01
sp|P15170|ERF3A	Eukaryotic peptide chain release factor GTP-binding subunit ERF3A GN = GSPT1	0.6628	< 0.05
sp|P04792|HSPB1	Heat shock protein beta-1 GN = HSPB1	0.6856	< 0.05
sp|Q9Y4E8|UBP15	Ubiquitin carboxyl-terminal hydrolase 15 GN = USP15	0.7569	< 0.05
sp|Q15057|ACAP2	Arf-GAP with coiled-coil, ANK repeat and PH domain-containing protein 2 GN = ACAP2	0.7614	< 0.05
sp|P05362|ICAM1	Inetercellular adhesion molecule 1 GN = ICAM1	0.7692	< 0.05
sp|P68402|PA1B2	Platelet-activating factor acetylhydrolase IB subunit beta GN = PAFAH1B2	1.2835	< 0.01
sp|P07900|HS90A	Heat shock protein HSP 90-alpha GN = HSP90AA1	1.3067	< 0.05
sp|P45973|CBX5	Chromobox protein homolog 5 GN = CBX5	1.3255	< 0.05
sp|Q14C86|GAPD1	GTPase-activating protein and VPS9 domain-containing protein 1 GN = GAPVD1	1.3355	< 0.05
sp|Q93009|UBP7	Ubiquitin carboxyl-terminal hydrolase 7 GN = USP7	1.4723	< 0.05
sp|P19338|NUCL	Nucleolin GN = NCL	1.5438	< 0.05
sp|P10599|THIO	Thioredoxin GN = TXN	4.4589	< 0.05
sp|P49585|PCY1A	Choline-phosphate cytidylyltransferase A GN = PCYT1A	100.0000	< 0.05

To identify cytoplasmic signaling networks potentially regulated by R-Ras subfamily proteins, we performed an Ingenuity Pathway Analysis (IPA) with the population of proteins whose levels changed (|log_2_(H:L)|> 0.33) in response to induction of DN R-Ras expression. These analyses indicated that DN R-Ras induction caused changes in thirteen networks distinct from those affected by DN H-Ras; the top seven ranked networks are indicated in Fig. 4b and Table 2. Notably, this IPA analysis identified two networks involved in the control of cellular movement via their effects on the dynamics of microtubules, actin and other cytoskeletal elements (Table 2, Additional file 6: Fig. S5). Networks regulating vesicular transport, amino acid and protein biosynthesis, RNA trafficking and DNA synthesis were also more prominently altered by DN R-Ras than by DN H-Ras, as were proteins involved in lipid metabolism (data not shown). Considered collectively, these findings indicate that R-Ras subfamily proteins regulate some signaling pathways distinct from those regulated by classic Ras proteins. The observation that some of these pathways potentially regulate cell motility and morphology suggests that one or more of them is essential for R-Ras mediated regulation of MPNST cell migration and invasion.

**Table 2:**
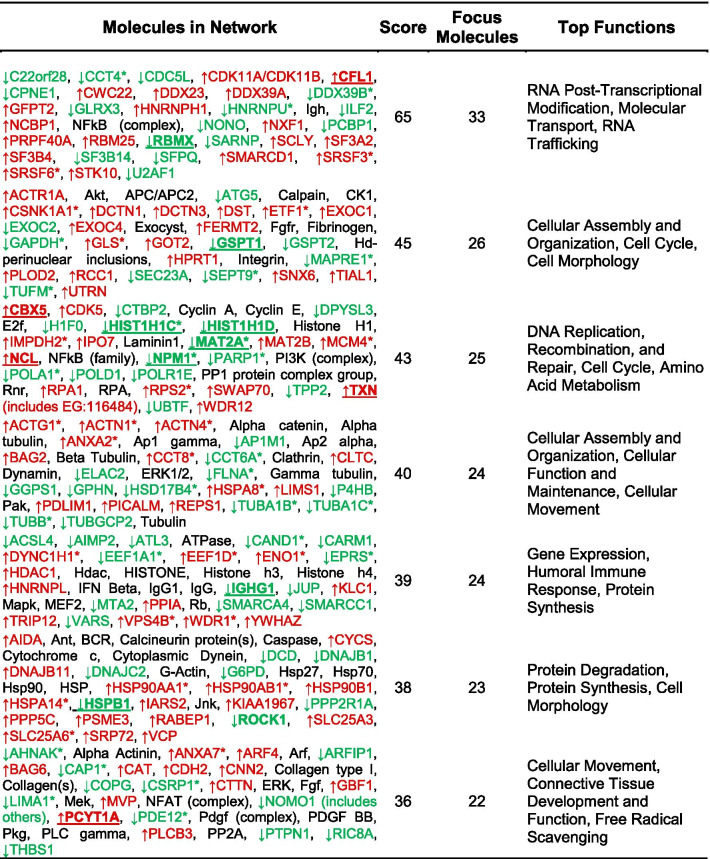
DN R-Ras IPA networks

Red gene symbols represent proteins which showed increased binding to the IMAC column following DN R-Ras induction, while green gene symbols indicate proteins with decreased binding

### R-Ras protein(s) mediate migration and cytoskeletal changes via ROCK1 activation

**Fig. 5:**
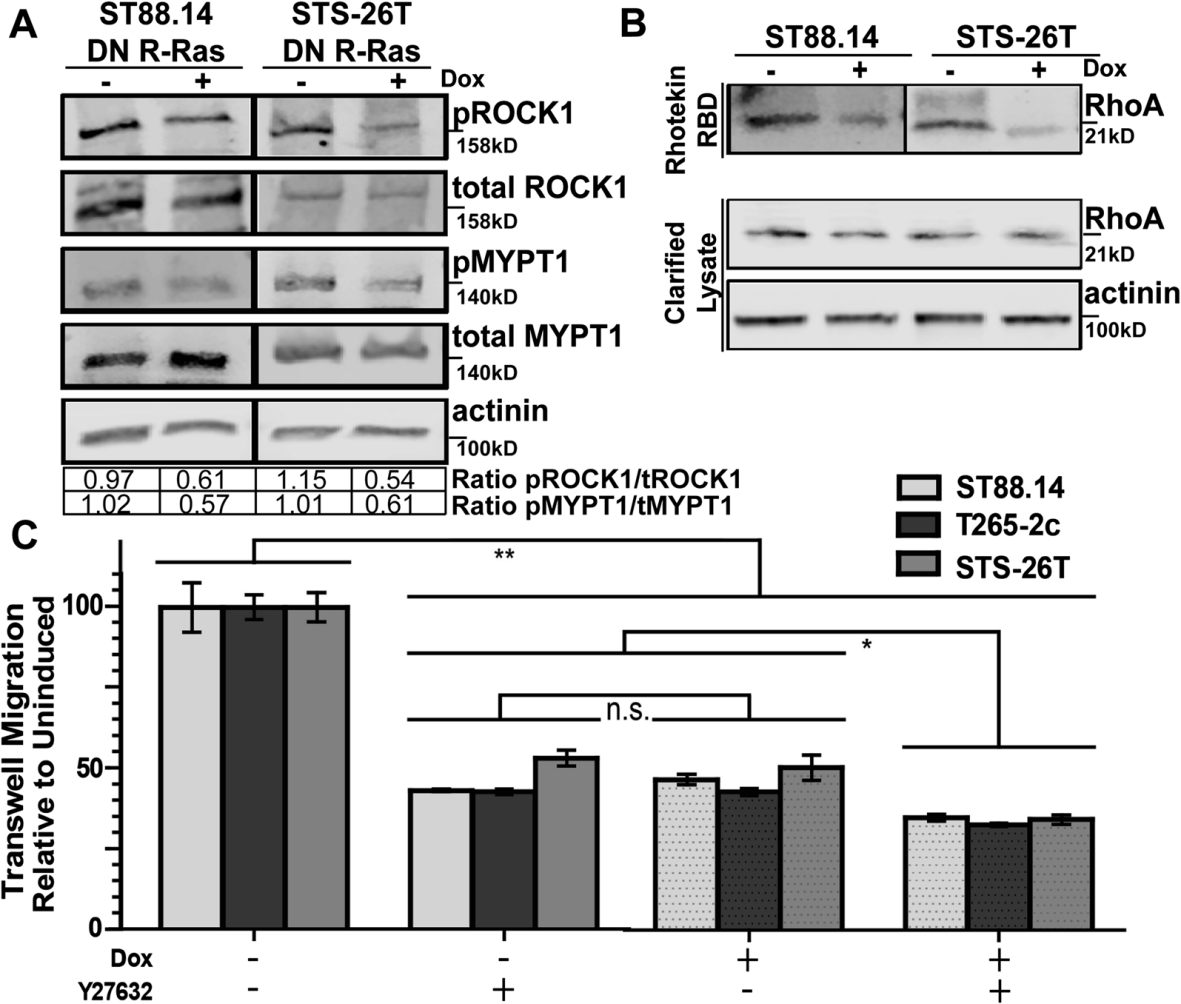
DN R-Ras inhibits phosphorylation of ROCK1 and MYPT1 and activation of RhoA while ROCK inhibition, like DN R-Ras expression, impedes migration. **a** Levels of total and phosphorylated ROCK1 and MYPT1 in ST88-14 and STS-26T cells stably transfected with doxycycline-inducible plasmids expressing DN R-Ras in the presence (+) and absence (−) of DN R-Ras expression. Blots were probed with an anti-actinin antibody as a loading control. Quantification indicated below the blots represents the ratio of the normalized phosphorylated to total protein levels. **b** Rhotekin pull-down assays in ST88-14 and STS-26T cells stably transfected with doxycycline-inducible plasmids expressing DN R-Ras. Induction of DN R-Ras expression (+) decreases RhoA activation. **c** Induction of DN R-Ras expression and treatment with the ROCK1 inhibitor Y27632 similarly reduce the migration of ST88-14, STS-26T, and T265 cells in Transwell migration assays. A combination of DN R-Ras expression and Y27632 treatment produces an additional small, but statistically significant reduction in migration. **, *p*-value < .0001; *, *p*-value < .05

To validate our phosphoproteomic data and expand our mechanistic insight into R-Ras mediated cellular processes, we examined the effects that DN R-Ras exerts on the actin cytoskeleton and the phosphorylation of ROCK1, a prominent hit in our studies. To assess the level of ROCK1 phosphorylation with and without DN R-Ras expression, we performed western blots for phosphorylated and total levels of ROCK1, quantified the intensity of each band and compared their relative levels. We found that phosphorylated ROCK1 levels were decreased following induction of DN R-Ras expression in ST88-14 (ratio ± standard deviation 0.97 ± 0.046 in uninduced cells versus 0.61 ± 0.079 in induced cells) and STS-26T (1.15 ± 0.11 uninduced versus 0.54 ± 0.11 induced) cells (Fig. 5a). To verify that this change was associated with decreased ROCK1 activity, we assessed the levels of phosphorylated and total MYPT1, a known downstream effector or ROCK1, following induction of DN R-Ras expression. We found that the phosphorylation of MYPT1 was also reduced following induction of DN R-Ras expression in ST88-14 (1.02 ± 0.028 uninduced versus 0.57 ± -0.03 induced) and STS-26T (1.01 ± 0.18 uninduced versus 0.61 ± 0.069 induced) cells (Fig. 5a). To determine if R-Ras regulation of ROCK1 phosphorylation was mediated by RhoA, a known regulator of cytoskeletal reorganization, we performed pulldown assays with Rhotekin Rho-binding domain (RBD), which binds activated RhoA. These assays showed that the expression of the DN R-Ras construct led to a decrease in RhoA activation, in keeping with decreased ROCK1 phosphorylation and activity in these cells (Fig. 5b).

We next compared the effect that DN R-Ras expression exerts on the migration of MPNST cells compared to the effects of the ROCK inhibitor Y27632. Using Transwell migration assays in ST88-14, T265-2c and STS-26T MPNST cells lines, we found that both DN R-Ras expression and Y27632 treatment resulted in significantly significant decreases in migration (*p* < 0.0001; Fig. 5c). Combining treatment with Y26732 with DN R-Ras expression produced an additional modest, but still statistically significant reduction in the migration of all three MPNST cell lines (Fig. 5c). These findings suggest that R-Ras subfamily members promote MPNST migration, at least in part, via their effects on ROCK1. However, the observation that Y26732 treatment and DN R-Ras expression further reduce migration also suggests that there are additional signaling pathways that are utilized by R-Ras subfamily members to promote MPNST migration.

**Fig. 6:**
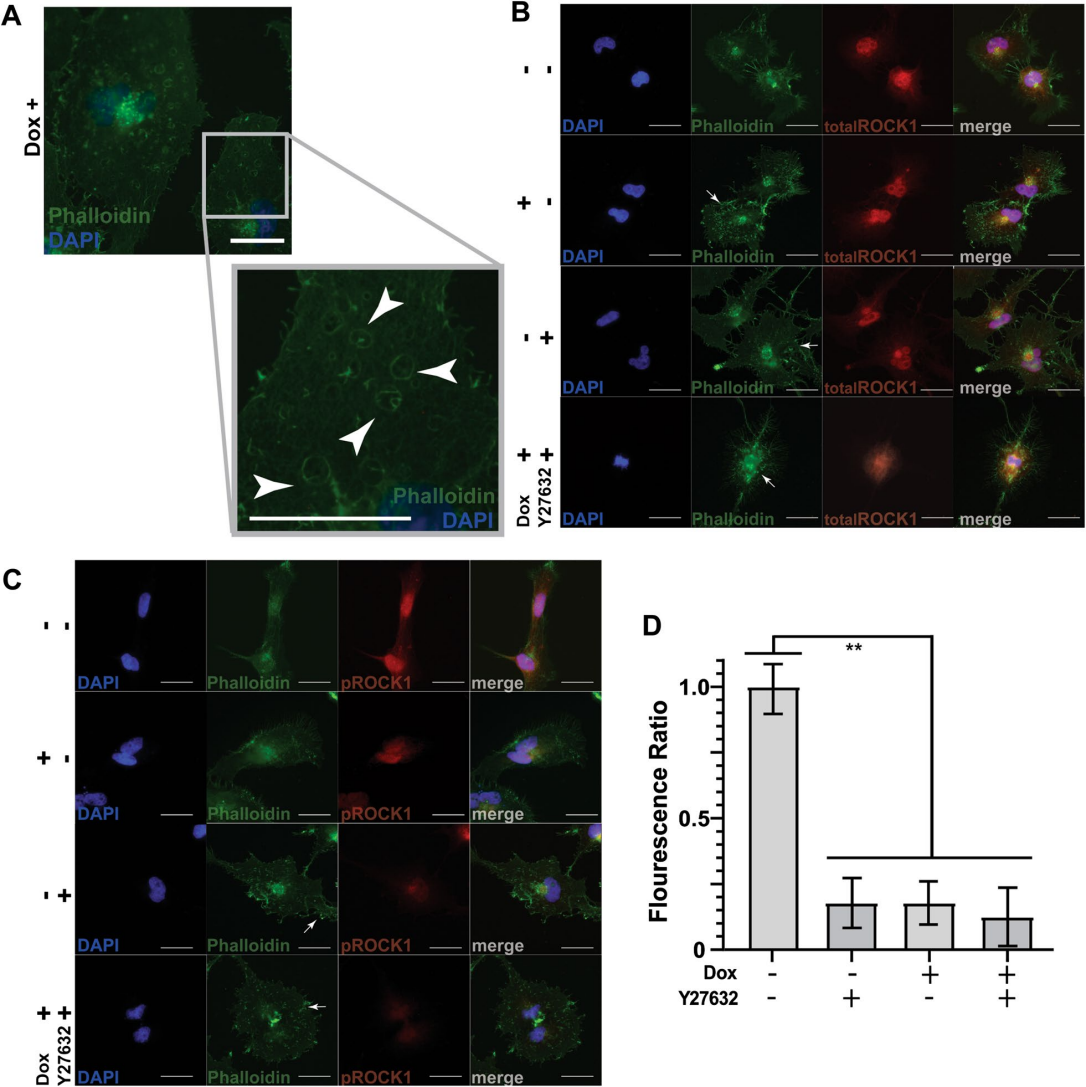
DN R-Ras decreases phosphorylation of ROCK1 and alters migration and cell morphology. **a** Effect of DN R-Ras expression on the actin cytoskeleton in ST88-14 cells as assessed by phalloidin staining. The enlarged image demonstrates actin-rimmed vesicular structures (white arrows) that accumulate in ST88-14 cells following induction of DN R-Ras expression. **b**, **c** Total ROCK1 (**b**; red) or phospho-ROCK1 immunoreactivity (**c**; red) in ST88-14 MPNST cells in the presence (+) or absence (−) of DN R-Ras expression and treatment with vehicle (−) or the ROCK inhibitor Y27632 (+). Cells were counterstained with phalloidin counterstain (green) to demonstrate cell morphology as highlighted by the actin cytoskeleton and bisbenzimide (blue) to label nuclei. Arrows denote notable changes in the actin cytoskeleton, including a loss of filamentous structures and the accumulation of actin-rich puncta (white arrows) following induction of DN R-Ras expression or Y27632 treatment. A non-immune isotype matched primary antibody was used as a negative control. Scale bars = 25 μm. **d** Semi-quantitative analysis of normalized total and phosphorylated ROCK1 levels relative to uninduced or untreated controls. **, *p*-value < 0.0001

To identify potential R-Ras mediated subcellular localization changes of pROCK1, we immunostained ST88-14 cells stably transfected with the doxycycline-inducible DN R-Ras vector with antibodies that recognize either total ROCK1 or ROCK1 phosphorylated on amino acids T^455^ and S^456^. In the absence of DN R-Ras expression, actin, as detected by phalloidin staining, was concentrated in the perinuclear region, with actin also evident in scattered puncta on the cell membrane and in thin protrusions from the cellular surface (Fig. 6a, arrows). Induction of DN R-Ras expression resulted in an increase in the number of actin-rich puncta, many of which were on the cell membrane, and more prominent protrusion of cellular extensions. Cells expressing DN R-Ras also contained numerous actin-rimmed vacuoles that were not present in the absence of DN R-Ras expression (Fig. 6a, arrows). Immunostains for total ROCK1 demonstrated that the majority of ROCK1 immunoreactivity was similarly localized in the perinuclear region (Fig. 6b) and that the expression of ROCK1 was unaffected by the expression of DN R-Ras; immunoblot analyses similarly showed no change in ROCK1 expression levels following the induction of DN R-Ras expression (Additional file 7: Fig. S6). Treatment of these cells with Y27632, similarly had no effect on the level of expression of ROCK1 in the presence or absence of DN R-Ras expression. Y27632 also affected the actin cytoskeleton, triggering the extension of numerous protrusions and increasing the number of membrane-associated actin-rich puncta (6A–C; arrows). In combination with DN R-Ras expression, Y27632 generated even larger numbers of actin-rich puncta, suggestive of cell stress (6B, C; arrows).

The distribution of phospho-ROCK1 in uninduced ST88-14 cells was similar to that of total ROCK1 (Fig. 6c compared to Fig. 6b). However, following the induction of DN R-Ras expression, phospho-ROCK1 immunoreactivity was reduced (Fig. 6c, d). Treatment of these cells with Y26732 similarly reduced phospho-ROCK1 immunoreactivity. Treatment of ST88-14 cells with Y26732 in the presence of DN R-Ras expression resulted in a nearly complete loss of phospho-ROCK1 immunoreactivity. A semi-quantitative analysis measuring cytoplasmic fluorescence intensity confirmed that the relative ratio of phosphorylated to total ROCK1 was decreased uniformly in cells treated with the ROCK1 inhibitor and following induction of DN R-Ras expression, validating our phosphoproteomic screen (Fig. 6d).

**Fig. 7:**
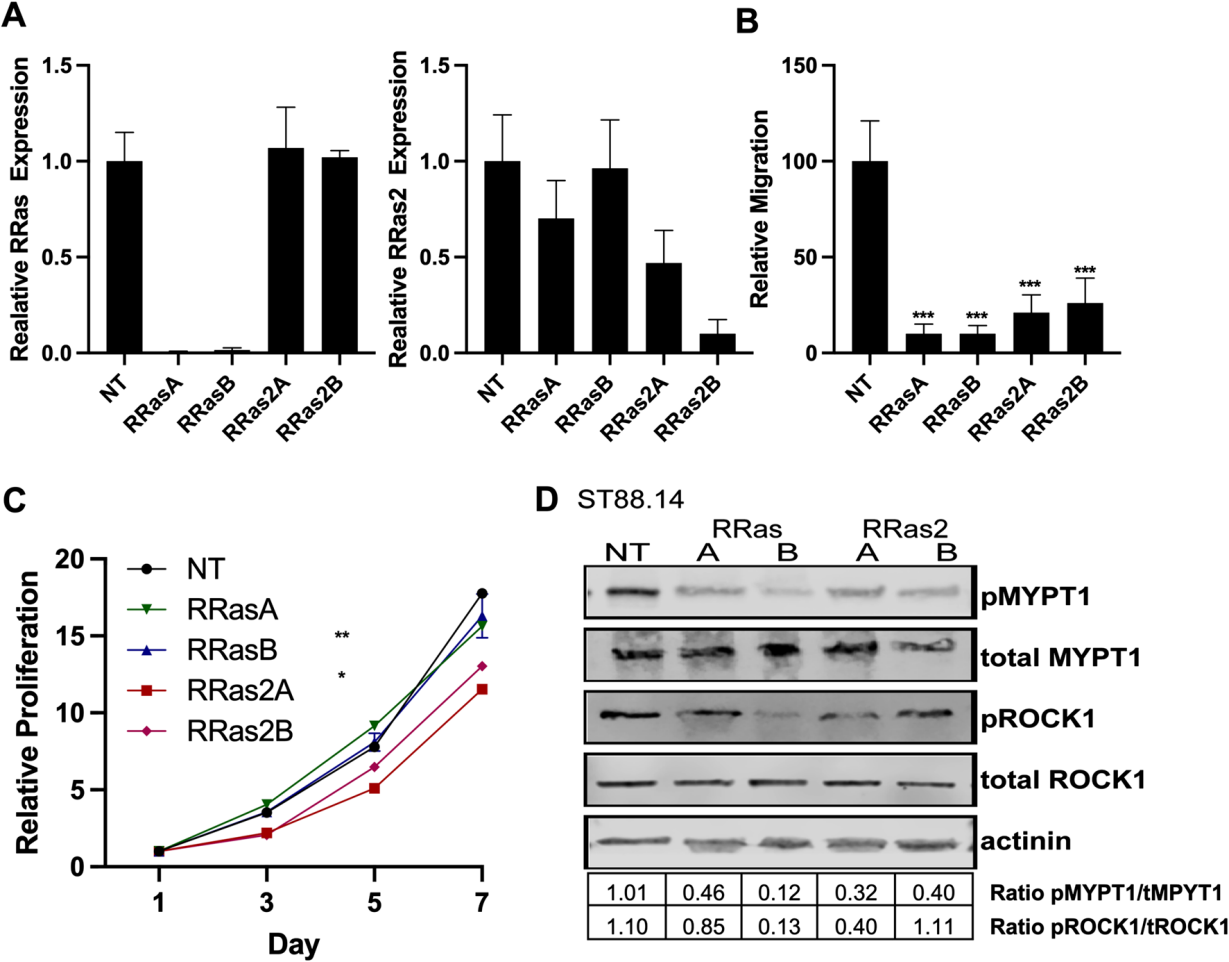
*RRAS* and *RRAS2* differentially promote proliferation and migration in ST88-14 cells. **a** Real-time PCR assays for *RRAS* (left panel) and *RRAS2* (right panel) mRNAs in ST88-14 cells transduced with lentiviruses expressing a non-targeting control shRNA (NT), *RRAS* shRNAs (RRasA, RRasB) or *RRAS2* shRNAs (RRas2A, RRas2B). **b** *RRAS* and *RRAS2* knockdown both potently inhibit the migration of ST88-14 MPNST cells. **c** *RRAS2*, but not *RRAS*, knockdown inhibits the proliferation of ST88-14 cells. **d** Both *RRAS* and *RRAS2* knockdown inhibit the phosphorylation of MYPT1 and ROCK1. Quantification indicated below the blots represents the ratio of the normalized phosphorylated to total protein levels

Our findings with the DN R-Ras construct do not distinguish between R-Ras and R-Ras2 functions. To determine whether R-Ras or R-Ras2 action affected the migration and proliferation of MPNST cells, we knocked down the expression of R-Ras or R-Ras2 in ST88-14 cells using shRNAs. As we did not have an antibody specific for R-Ras2 (Additional file 3: Fig. S2), we validated the level of knockdown using real time-PCR. We found that the two R-Ras shRNAs that we tested nearly completely ablated *RRAS* mRNA expression, while having no effect on the levels of *RRAS2* mRNA (Fig. 7a, left). The R-Ras2 shRNAs knocked down RRAS2 mRNA expression 50–90% (Fig. 7a, right), while having no statistically significant effect on *RRAS* mRNA levels. Upon performing transwell migration assays with ST88-14 cells in which RRAS or RRAS2 expression had been knocked down, we found that loss of either *RRAS* or *RRAS2* potently impaired MPNST migration (Fig. 7b). In contrast, knockdown of *RRAS* expression had no effect on the proliferation of ST88-14 cells, while *RRAS2* knockdown produced a modest, but statistically significant reduction in the proliferation of these cells (Fig. 7c). This aligns with literature suggesting that R-Ras2, but not R-Ras, has oncogenic properties similar to those of the classic Ras proteins [50].

To determine whether R-Ras or R-Ras2 was responsible for activating ROCK1, we assessed the levels of phosphorylated and total ROCK1 and MYPT1 in ST88-14 cells transfected with R-Ras and R-Ras2 shRNAs. We found that neither RRAS or RRAS2 knockdowns altered the levels of total ROCK1 and MYPT1 (Fig. 7d). In contrast, knockdown of both *RRAS* and *RRAS2* decreased the levels of phosphorylated ROCK1 and MYPT1 in these cells, with the exception of one *RRAS2* shRNA which showed no decrease in pROCK but did show a decrease in pMYPT1 (Fig. 7d), suggesting that both of these R-Ras subfamily members promote the activation of the RhoA/ROCK1/MYPT1 signaling cascade.

## Discussion

We have found that a subset of R-Ras subfamily proteins is expressed in *NF1*-null and sporadic MPNST cell lines. Although M-Ras was typically undetectable in the MPNST lines we examined, uniform expression of R-Ras2 protein was evident in these lines with R-Ras also present in a major subset of the lines. We found that exogenous R-Ras and R-Ras2 are both activated in *NF1*-null ST88-14 MPNST cells (a line that normally expresses both R-Ras and R-Ras2), based on the finding that these proteins bind to the Raf-1 Ras binding domain. Although the interaction of R-Ras and R-Ras2 with full-length Raf-1 is controversial [16, 53–58] and may be cell-type dependent, our observations are consistent with previous reports demonstrating that activated R-Ras and R-Ras2 bind to a truncated Raf-1 Ras binding domain [16, 54]. To our knowledge, this is the first direct demonstration that R-Ras and R-Ras2 are activated and functional in neurofibromin-null MPNST cells. However, the fact that R-Ras and R-Ras2 are activated in *NF1*-null MPNST cells is consistent with earlier studies indicating that neurofibromin inactivates these proteins in vitro [27] and implicating R-Ras proteins in the enhanced migration of *Nf1*^−/−^ non-neoplastic Schwann cells [40]. We do not yet know whether both R-Ras and R-Ras2 are required for MPNST pathogenesis. However, we did find that three MPNST cell lines—YST-1, 90-8 and NMS2—had no detectable R-Ras expression, indicating that R-Ras2 is the only functional R-Ras protein in these cells. Our observation that R-Ras2 is uniformly present in MPNST cells while R-Ras is more variably expressed suggests that R-Ras2 is likely to be particularly important in the pathogenesis of some MPNSTs. However, in ST88-14 MPNST cells, we found that both *RRAS* and *RRAS2* are essential for migration, suggesting that R-Ras plays a role in the pathogenesis of a subset of MPNSTs.

Our finding that DN R-Ras, unlike DN H-Ras, reduced the migration of MPNST cells is consistent with previous observations in *Nf1*^−/−^ Schwann cells [40] and suggests that R-Ras subfamily members function similarly in neoplastic Schwann cells and their non-neoplastic counterparts. However, our finding that DN R-Ras expression and *RRAS2* knockdown both inhibit MPNST mitogenesis is, to the best of our knowledge, the first evidence implicating R-Ras2 in the proliferation of MPNST cells. Of note, we also found that that the migration of STS-26T cells, a sporadic line which maintains intact neurofibromin and does not show Ras hyperactivation [52], was inhibited by DN R-Ras to an extent similar to that seen in our *NF1*-null cells, while their proliferation was relatively resistant to DN R-Ras. To explain this, we considered the possibility that STS-26T cells might have acquired an activating mutation in a single R-Ras subfamily member (or a gene encoding a protein directly downstream of R-Ras/R-Ras2), thereby rendering them resistant to DN R-Ras mediated inhibition of proliferation but not migration. However, we have performed whole exome sequencing on STS-26T cells and have found no evidence of such mutations (Longo et al., unpublished observations). In the future, it will be of interest to see whether the DN R-Ras mutant has similar effects in other sporadic MPNST cell lines and to determine how R-Ras proteins are activated in MPNST cells with intact neurofibromin expression.

**Fig. 8:**
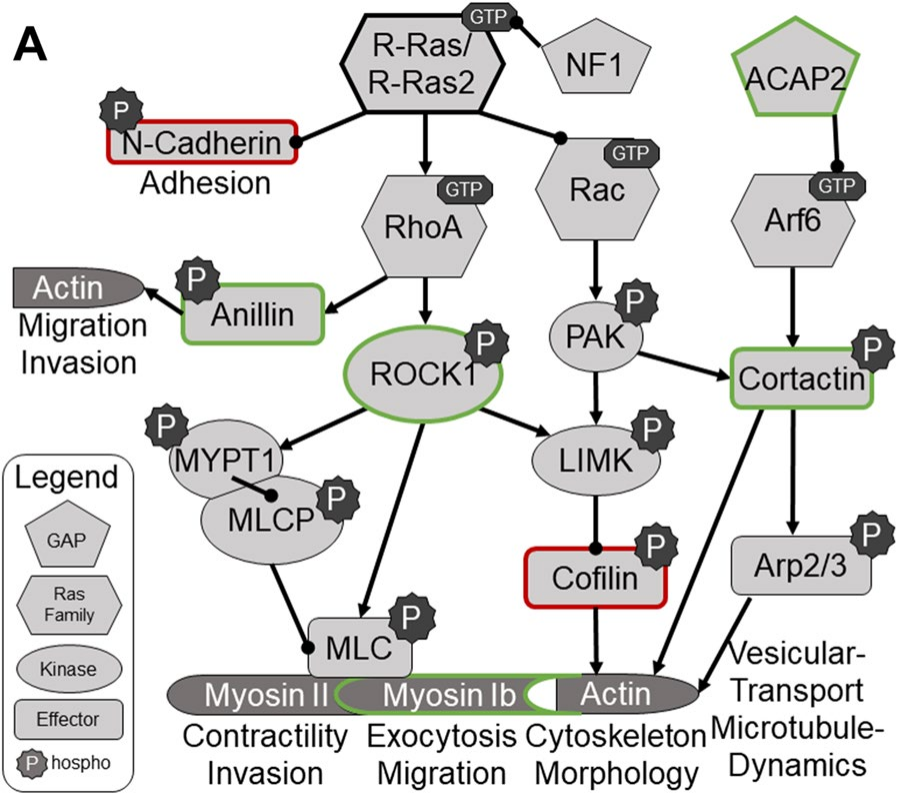
Signaling pathways potentially affected by R-Ras subfamily signaling as implicated by changes in the phosphoproteome triggered by DN R-Ras expression. Proteins rimmed in green have increased phosphorylation following activation of R-Ras and/or R-Ras2, while proteins rimmed in red show reduced phosphorylation following activation of R-Ras and/or R-Ras2

The effects of DN R-Ras on MPNST migration, considered together with our previous findings [33], suggest that classic Ras proteins control a set of cellular signaling cascades distinct from those controlled by R-Ras proteins. Using a global phosphoproteomics approach, we examined the changes in protein phosphorylation that occurred in response to DN R-Ras expression. We found that inhibition of R-Ras protein signaling altered the phosphorylation of proteins in multiple signaling cascades that were not affected by the expression of DN H-Ras. Since DN R-Ras, unlike DN H-Ras, inhibited migration and invasion, we focused particularly on signaling cascades that have been previously implicated in cellular movement and changes in cellular morphology. These analyses demonstrated multiple hits within relevant and interconnected pathways, indicative of cohesive downstream effects of DN R-Ras expression. We recognize that the networks potentially controlling migration and invasion are complex and that spatial and temporal regulation of protein expression and phosphorylation also plays a key role in their regulation. With these caveats in mind, we evaluated the phosphorylation changes that followed the induction of DN R-Ras expression and were able to construct an initial outline of key signaling pathways downstream of R-Ras proteins (Fig. 8).

Alterations in the phosphorylation of proteins in cytoplasmic signaling cascades that regulate cytoskeletal structure were particularly prominent in our analyses. This included significant changes in the phosphorylation of proteins controlling actin dynamics—proteins such as ROCK1, cofilin and cortactin; cofilin inhibition downstream of Rho and ROCK1 activation facilitates the polymerization of G-actin and promotes morphological changes [59–63]. Other top networks showed alterations in the phosphorylation of microtubule subunits (e.g., tubulin alpha-1B and -1C chains) and microtubule binding proteins (e.g., dynactin subunits 1 and 3, kinesin light chain 1), a collection of molecules implicated in cellular movement. This suggests that DN R-Ras reduces migration by impacting both actin dynamics and microtubule-dependent phenomenon such as selective microtubule stabilization, the reorientation of the microtubule-organizing center (MTOC) or directed vesicular transport towards the leading edge of migrating cells [64]. Although R-Ras has previously been implicated in microtubule stability in hippocampal neuron growth cones [65, 66] through its effects on PI3K/Akt signaling, Akt signaling did not appear to be affected by DN R-Ras expression in our experiments (Additional file 5: Fig. S4) suggesting that in MPNSTs R-Ras signaling predominantly activates the Rho pathway. We would also note that previous studies have linked the activation of integrins, which function both upstream [63] and downstream [67] of microtubule dynamics during migration, to the promotion of migration by activated R-Ras [62, 68, 69]. As our phosphoproteomics protocol was geared towards examining cytoplasmic proteins rather than integral membrane proteins, we were not able to directly assess impaired integrin activation by DN R-Ras. In the future, it will be of interest to examine the effect that altered R-Ras and R-Ras2 signaling exert on integrins.

Our phosphoproteomics data also suggested a potential role for Arf6, a RAS superfamily protein involved in vesicular trafficking, in coordinating R-Ras dependent effects on microtubule and integrin function. The Arf6 GTP-binding protein promotes migration by enhancing post-endocytic trafficking of β1-integrin [19, 35, 62, 67], Cdc42 [70] and Rac1 [62, 71, 72] to the leading edge of cells in a microtubule- and AP2-dependent manner. As Arf6 is regulated by GTP/GDP binding, its phosphorylation would not be expected to change based on its activation state, and indeed, we did not observe changes in phosphorylated Arf6 levels following DN R-Ras induction. However, we did find that phosphorylation of the phosphoinositide-activated Arf6 GTPase activating protein, ACAP2, was significantly reduced following DN R-Ras induction; ACAP2 was a hit in a signaling network mediating molecular transport. Interestingly, Arf6 works with RhoA, the main activator of ROCK1, to generate tumor-derived microvesicles, suggesting a potential role for the R-Ras family in this process [73]. ROCK-dependent actin remodeling has also been implicated as a negative regulator of autophagosome formation [74]. This earlier finding is consistent with our observation that treatment of MPNST cells with the ROCK inhibitor Y27632 results in the accumulation of abnormal vesicular structures; the presence of abnormal vesicular structures in MPNST cells following the induction of DN R-Ras expression is consistent with our finding that ROCK1 phosphorylation and activation, as assessed by the phosphorylation of MYPT1 is reduced in these same cells; DN R-Ras also inhibits the activation of RhoA, the activator of ROCK1, in MPNST cells, which further clarifies the biological role of this R-Ras effector molecule in MPNST pathogenesis. Further, ROCK1 can activate LIMK, which inhibits cofilin, leading to actin cytoskeletal remodeling and tumor cell invasion [75–77]. Thus, these findings provide important new evidence regarding how R-Ras subfamily proteins regulate cellular morphology, migration, invasion and vesicular trafficking/structure. These observations also provide a foundation for further research further detailing the intricacies of how R-Ras proteins regulate migration, morphology, invasion, vesicular trafficking and potentially autophagosome formation.

When we attempted to distinguish between R-Ras and R-Ras2 effects on migration, we found that knockdown of the mRNAs encoding either protein resulted in decreases in the migration of ST88-14 MPNST cells. We also found that both *RRAS* and *RRAS2* contribute to the activation of the RhoA/ROCK1/MYPT1 signaling cascade, a pathway that is critically important for the migration of MPNST cells. However, the fact that ablation of *RRAS* is not compensated for by *RRAS2* (and vice versa) raises the question of whether both of these proteins have additional, distinct effects on other signaling cascades that are also essential for MPNST migration. Alternatively, it may be that R-Ras and R-Ras2 regulate the same pro-migratory signaling pathways and that the joint expression of both proteins is required to reach a threshold of R-Ras subfamily signaling sufficient to drive migration. This latter possibility would be consistent with the fact that several of the MPNST cell lines we examined expressed only R-Ras2. In future studies, it will interesting to ablate the expression of R-Ras and R-Ras2 and then determine whether these proteins differ in their ability to regulate signaling cascades that are required to promote the migration of MPNST cells.

## Conclusions

We demonstrated that inhibition of R-Ras family proteins leads to decreased proliferation, migration, and invasion, indicating these proteins have actions distinct from (migration and invasion) and shared with (proliferation) classic Ras proteins. Phosphoproteomic analyses identified 13 signaling pathways specifically regulated by R-Ras proteins, many of which are suggested to regulate cellular morphology and migration. Exploration into the cytoplasmic signaling cascade networks downstream of the R-Ras subfamily in MPNSTs warrants further investigation as potential novel therapeutic targets.

## Supplementary Information

**Additional file 2**. **Figure S1** (A) immunoblot for neurofibromin protein in the MPNST cell lines used in this study. Blots were probed with an anti-actinin antibody to compare loading. (B) NF1 mutational status of the MPNST cell lines. (C) RT-PCR assays for MRAS mRNA show that these transcripts are only present in S462 cells. (D) Cluster analysis comparing the relatedness of the R-Ras and classic Ras protein subfamilies. (E) Expression of HA-tagged DN R-Ras in stably transfected ST88-14 cells with varying concentrations of doxycycline. Expression is maximally induced with 2 µg/ml doxycycline. (F) Relative expression levels of HA-tagged DN R-Ras in ST88-14 cells at various time points after addition of 2µg/ml doxycycline. Expression is maximally induced 48hr after the addition of doxycycline.

**Additional file 3**. **Figure S2** Determination of R-Ras antibody specificity. ST88-14 cells were transiently transfected with Myc-tagged dominant negative H-Ras or wild-type R-Ras or R-Ras2. Lysates of these cells were immunoblotted for R-Ras isoform expression using antibodies directed against R-Ras (Abnova H00006237_M01, 1:10,000) or R-Ras2 (Abnova H000022800_M01, 1:10,000; sc-833 1:1000; sc-81931 1:10,000; R&D Systems AF3605 1:1000; Abcam 96307 1:10,000). A sixth R-Ras2 antibody (sc-166232, 1:100) failed to recognize a band at the appropriate molecular weight (data not shown). Endogenous Ras (which migrates ∼5kD below Myc-tagged isoforms) is not shown.

**Additional file 4**. **Figure S3** Phosphoprotein enrichment of stable-isotope labeled doxycycline-inducible cell lines. Immunoblotting for representative phosphoproteins verifies phosphoprotein enrichment. Induction of dominant negative mutants was verified by immunoblotting for the HA epitope. Antibody dilutions were as follows: p-Erk1/2 1:1200, HA epitope 1:50,000, GAPDH 1:100,000.

**Additional file 5**. **Figure S4** Sample MS and MS/MS spectra. MS spectra corresponding to the heavy- and light-labeled peptide LIFAGK from the ubiquitin-60S ribosomal protein L40 (sp|P62987|RL40_HUMAN) are shown. Relative peak intensities of all heavy- and light-labeled detected peptides are used to estimate relative quantitation of parent proteins. The MS/MS spectra of the LIFAGK peptide show the daughter *b*- and *y*-ions generated by collision with the neutral gas helium; these ions are used to confirm the peptide sequence.

**Additional file 6**. **Figure S5** Signalling pathways identified by IPA whose activation is modified by DN R-Ras expression.

**Additional file 7**. **Figure S6** Signalling effectors affected by DN R-Ras induction. (A) Expression of induced DN R-Ras ST88-14 MPNST cells compared to control, either uninduced DN R-Ras ST88-14 MPNST cells or induced GFP ST88-14 MPNST cells. R-Ras and R-Ras2 immunoblotting show the presence of the HA-tagged DN R-Ras as a thick band above the endogenous Ras/Ras2 levels. Immunoblots comparing the effect of DN R-Ras expression on cofilin, RhoA, pERK1/2, total ERK1/2, pAKT and total AKT are also shown. (B) Raf-1 RBD pulldown assays demonstrating the effect of DN R-Ras expression on the activation of R-Ras proteins.

**Additional file 8**

**Additional file 9**

**Additional file 10**

**Additional file 11**

## Data Availability

All data generated or analyzed during this study are included in this published article and supplementary information files.

## Abbreviations

MPNST: Malignant peripheral nerve sheath tumor
NF1: Neurofibromatosis type 1
DN: Dominant negative
ROCK1: Rho-associated, coiled-coil-containing protein kinase 1
IPA: Ingenuity pathway analysis
