# Supplementary figures and images for: R-Ras subfamily proteins elicit distinct physiologic effects and phosphoproteome alterations in neurofibromin-null MPNST cells
Source: Cell Commun Signal. 2021 Sep 16;19:95. doi: 10.1186/s12964-021-00773-4 (PMC8447793; doi:10.1186/s12964-021-00773-4)

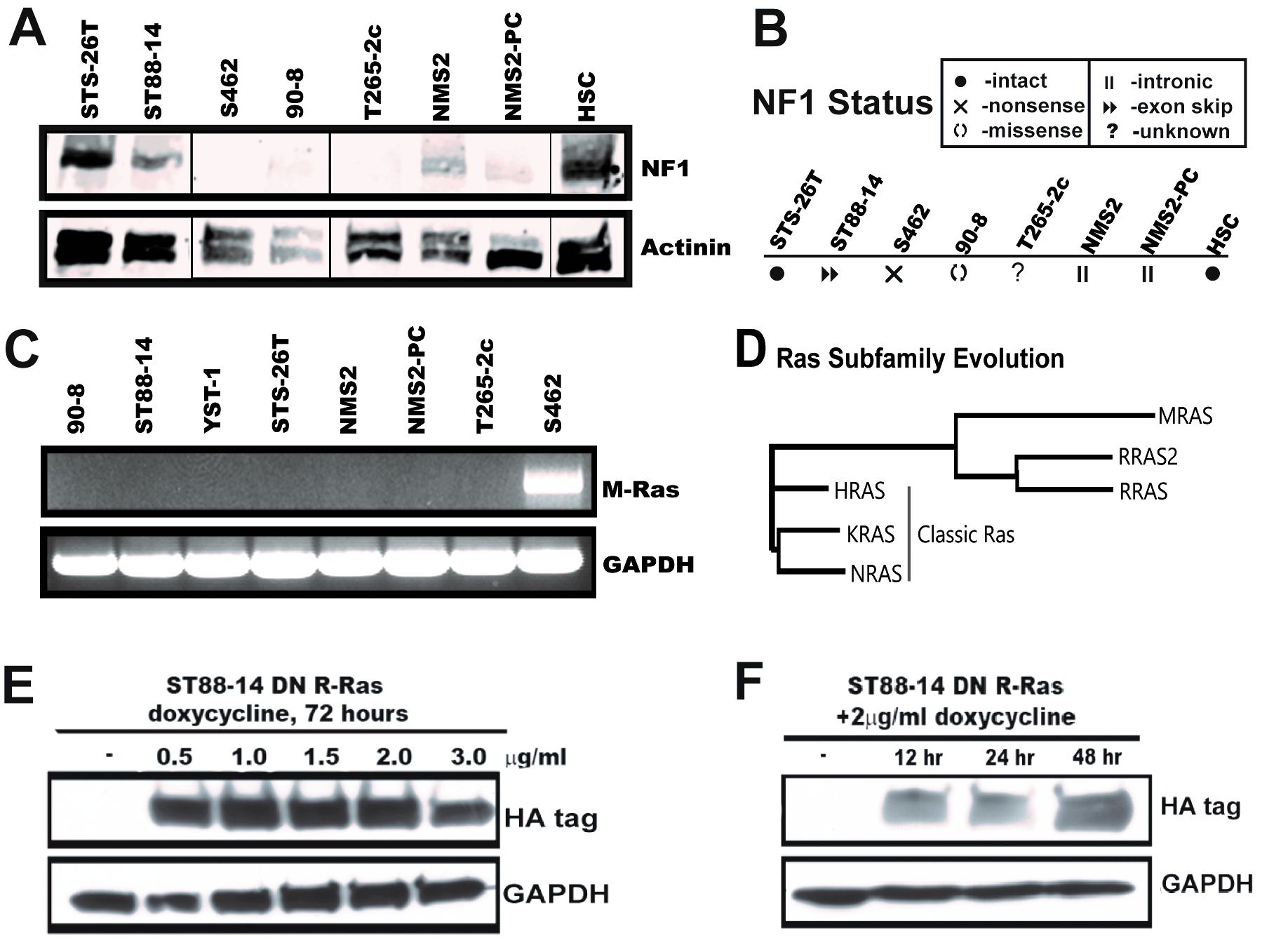

Supplement: Supplementary file 2 — Additional file 2. Figure S1 (A) immunoblot for neurofibromin protein in the MPNST cell lines used in this study. Blots were probed with an anti-actinin antibody to compare loading. (B) NF1 mutational status of the MPNST cell lines. (C) RT-PCR assays for MRAS mRNA show that these transcripts are only present in S462 cells. (D) Cluster analysis comparing the relatedness of the R-Ras and classic Ras protein subfamilies. (E) Expression of HA-tagged DN R-Ras in stably transfected ST88-14 cells with varying concentrations of doxycycline. Expression is maximally induced with 2 µg/ml doxycycline. (F) Relative expression levels of HA-tagged DN R-Ras in ST88-14 cells at various time points after addition of 2µg/ml doxycycline. Expression is maximally induced 48hr after the addition of doxycycline. [file 12964_2021_773_MOESM2_ESM.tif]

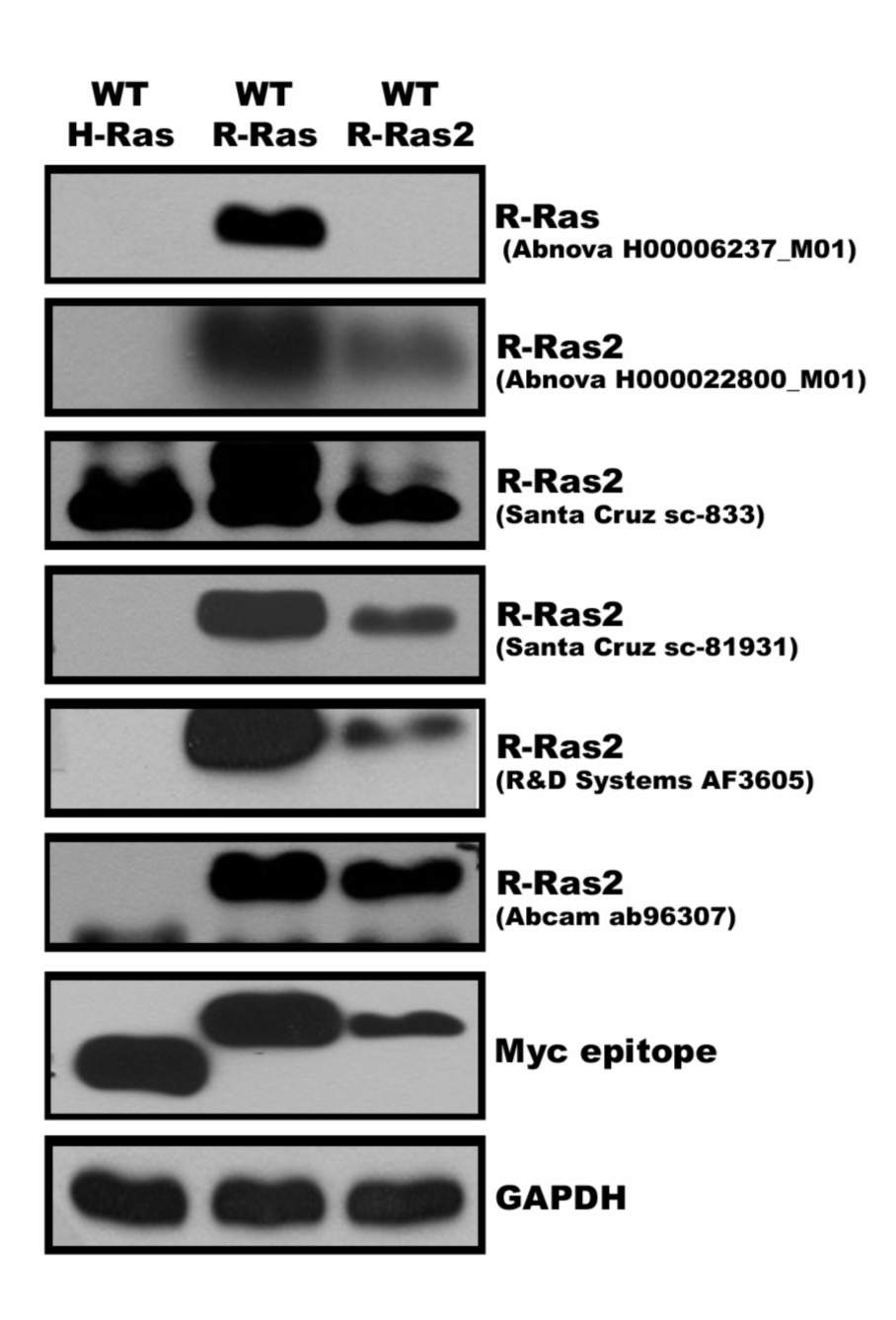

Supplement: Supplementary file 3 — Additional file 3. Figure S2 Determination of R-Ras antibody specificity. ST88-14 cells were transiently transfected with Myc-tagged dominant negative H-Ras or wild-type R-Ras or R-Ras2. Lysates of these cells were immunoblotted for R-Ras isoform expression using antibodies directed against R-Ras (Abnova H00006237_M01, 1:10,000) or R-Ras2 (Abnova H000022800_M01, 1:10,000; sc-833 1:1000; sc-81931 1:10,000; R&D Systems AF3605 1:1000; Abcam 96307 1:10,000). A sixth R-Ras2 antibody (sc-166232, 1:100) failed to recognize a band at the appropriate molecular weight (data not shown). Endogenous Ras (which migrates ∼5kD below Myc-tagged isoforms) is not shown. [file 12964_2021_773_MOESM3_ESM.tif]

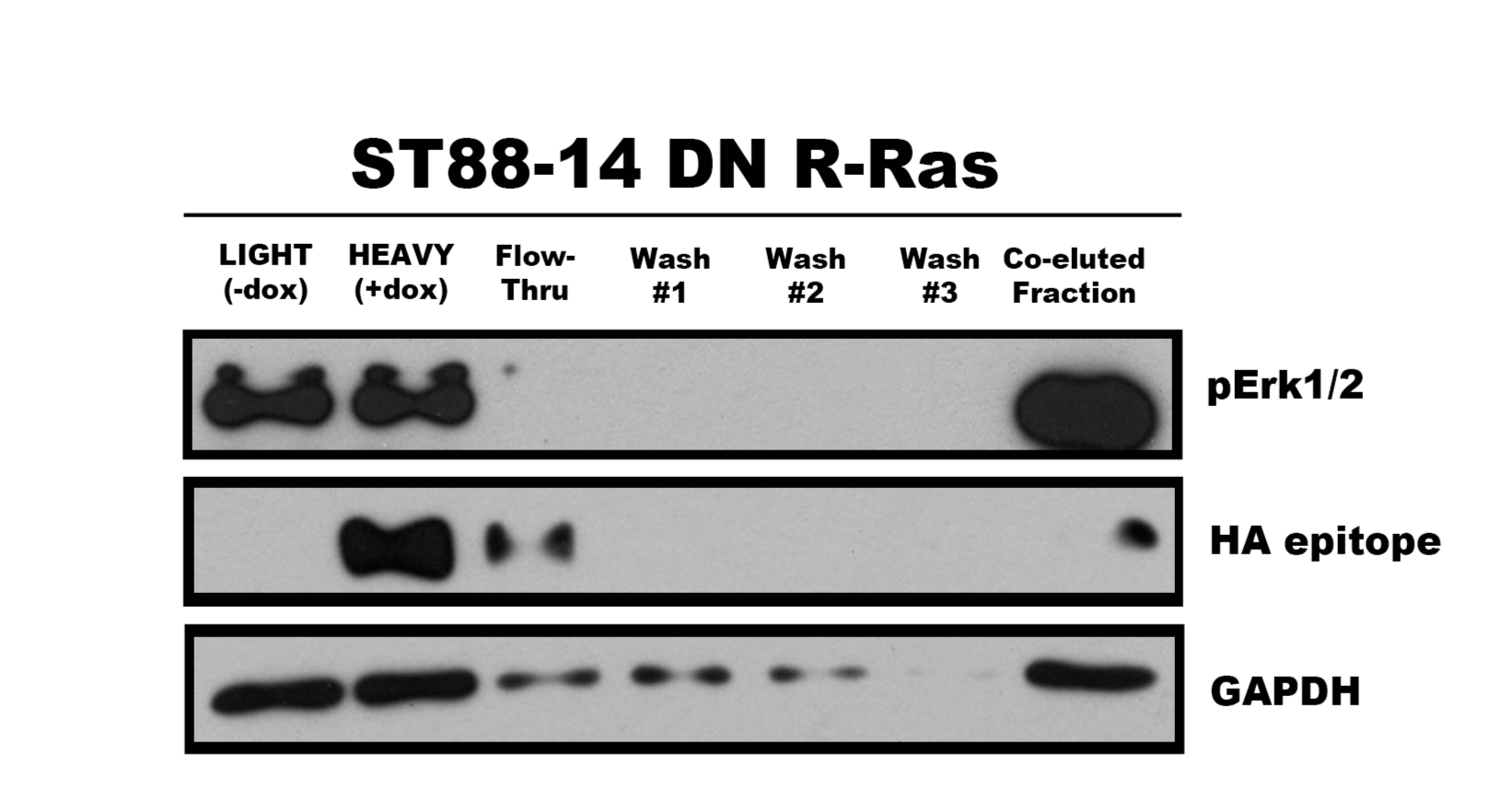

Supplement: Supplementary file 4 — Additional file 4. Figure S3 Phosphoprotein enrichment of stable-isotope labeled doxycycline-inducible cell lines. Immunoblotting for representative phosphoproteins verifies phosphoprotein enrichment. Induction of dominant negative mutants was verified by immunoblotting for the HA epitope. Antibody dilutions were as follows: p-Erk1/2 1:1200, HA epitope 1:50,000, GAPDH 1:100,000. [file 12964_2021_773_MOESM4_ESM.tif]

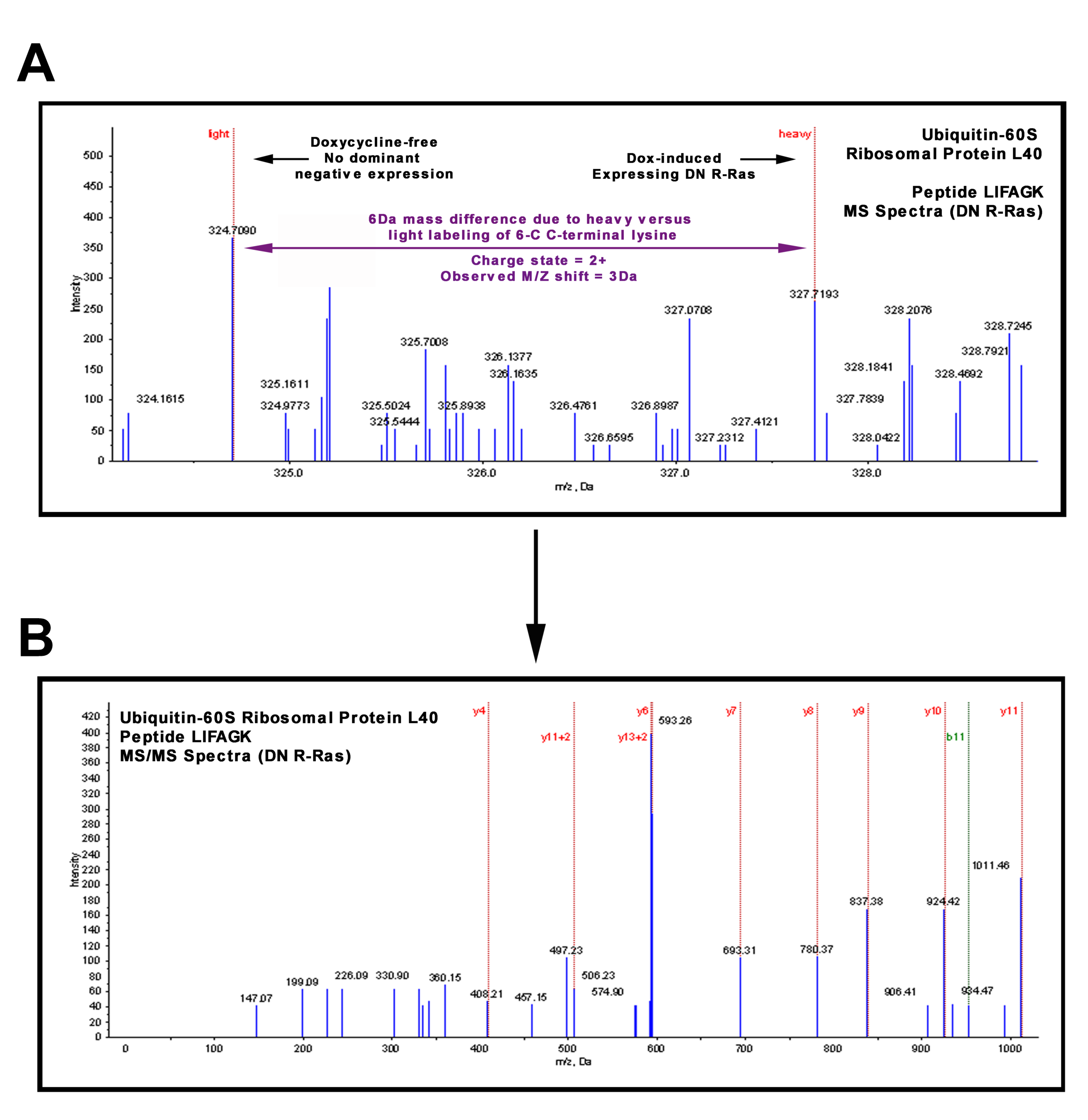

Supplement: Supplementary file 5 — Additional file 5. Figure S4 Sample MS and MS/MS spectra. MS spectra corresponding to the heavy- and light-labeled peptide LIFAGK from the ubiquitin-60S ribosomal protein L40 (sp|P62987|RL40_HUMAN) are shown. Relative peak intensities of all heavy- and light-labeled detected peptides are used to estimate relative quantitation of parent proteins. The MS/MS spectra of the LIFAGK peptide show the daughter b- and y-ions generated by collision with the neutral gas helium; these ions are used to confirm the peptide sequence. [file 12964_2021_773_MOESM5_ESM.tif]
