# Supplementary material for: R-Ras subfamily proteins elicit distinct physiologic effects and phosphoproteome alterations in neurofibromin-null MPNST cells
Source: Cell Commun Signal. 2021 Sep 16;19:95. doi: 10.1186/s12964-021-00773-4 (PMC8447793; doi:10.1186/s12964-021-00773-4)
Supplement: Supplementary file 8 — Additional file 8 [file 12964_2021_773_MOESM8_ESM.docx]

**Table S1: Antibodies**

| **Antibody Target** | **Vender** | **Catalog #** | **Application** | |
| --- | --- | --- | --- | --- |
| R-Ras | Abnova | H00006237_M01 | | IB |
| R-Ras2/TC21 | Abnova | H00022800_M01 | | IB |
| R-Ras2/TC21 | Santa Cruz Biotechnology | sc-833, sc-81931 | | IB |
| R-Ras2/TC21 | R&D Systems | AF3605 | | IB |
| R-Ras2/TC21 | Abcam | ab96307 | | IB |
| H-Ras | Santa Cruz Biotechnology | sc-520, sc-833 | | IB |
| H-Ras (Y132) | Abcam | ab32417 | | IB |
| M-Ras (N-19) | Santa Cruz Biotechnology | sc-8168 | | IB |
| HA-tag | Santa Cruz Biotechnology | sc-805 | | IB |
| Myc tag | Cell Signaling Technology | 2276 | | IB |
| GFP | Sigma-Aldrich | G1546 | | IB |
| GAPDH | Fitzgerald Industries Intl | RDI-TRK5G4-6C5 | | IB |
| Phospho-Erk1/2^Thr202/Tyr204^ | Cell Signaling Technology | 31925 | | IB |
| Erk1/2 | Cell Signaling Technology | 9108 | | IB |
| Cofilin(D59) | Cell Signaling Technology | 3318 | | IB |
| RhoA (67B9) | Cell Signaling Technology | 2117 | | IB |
| Phospho-AKT^Ser473^ | Cell Signaling Technology | 9271 | | IB |
| Phospho-AKT^Ser473^ | Cell Signaling Technology | 4051 | | IB |
| AKT | Cell Signaling Technology | 2966 | | IB |
| AKT | Cell Signaling Technology | 9272 | | IB |
| ROCK1 (C8S7) | Cell Signaling Technology | 4035 | | IB/ICC |
| Phospho-ROCK1^T455/S456^ | Abcam | ab203273 | | ICC |
| Rb mAb IgG XP^®^ | Cell Signaling Technology | 3900 | | ICC |
| HRP-conjugated  secondary antibodies | Jackson ImmunoResearch Laboratories | 109-035-003,  115-035-003 | | IB |
| IRDye® secondary  antibodies | Li-Cor | IRDye 800CW,  IRDye 680RD | | IB |
| Alexa-Fluor secondary antibodies | Thermo-Fisher | A-11036 | | ICC |
