# Supplementary material for: R-Ras subfamily proteins elicit distinct physiologic effects and phosphoproteome alterations in neurofibromin-null MPNST cells
Source: Cell Commun Signal. 2021 Sep 16;19:95. doi: 10.1186/s12964-021-00773-4 (PMC8447793; doi:10.1186/s12964-021-00773-4)
Supplement: Supplementary file 9 — Additional file 9 [file 12964_2021_773_MOESM9_ESM.docx]

**Table S2: Conventional PCR Primer Sequences**

| **Gene** | **Sequence** | **Direction** |
| --- | --- | --- |
| R-Ras | GCGCTCACCATCCAGTTCAT | FWD |
| R-Ras | CAGTCCTATTTTGTAACGGATTATGATC | REV |
| R-Ras2 | CGTGGACGAGGCTTTTGAG | FWD |
| R-Ras2 | TGGGAGCTCTTGTTCCTGGTA | REV |
| M-Ras | AATTGCCCCTTTTTCTGTTCC | FWD |
| M-Ras | TTATGGCCGAGGTCTCTGTTCTAC | REV |
