# Supplementary material for: R-Ras subfamily proteins elicit distinct physiologic effects and phosphoproteome alterations in neurofibromin-null MPNST cells
Source: Cell Commun Signal. 2021 Sep 16;19:95. doi: 10.1186/s12964-021-00773-4 (PMC8447793; doi:10.1186/s12964-021-00773-4)
Supplement: Supplementary file 10 — Additional file 10 [file 12964_2021_773_MOESM10_ESM.docx]

**Table S3: Real-Time PCR Primers**

| Gene | Catalog Number |
| --- | --- |
| R-Ras | Thermo Taqman Probes #4453320 |
| R-Ras2 | Thermo Taqman Probes #4448892 |
