# Supplementary material for: R-Ras subfamily proteins elicit distinct physiologic effects and phosphoproteome alterations in neurofibromin-null MPNST cells
Source: Cell Commun Signal. 2021 Sep 16;19:95. doi: 10.1186/s12964-021-00773-4 (PMC8447793; doi:10.1186/s12964-021-00773-4)
Supplement: Supplementary file 11 — Additional file 11 [file 12964_2021_773_MOESM11_ESM.docx]

**Table S4: Plasmids purchased from cDNA Resource Center**

| **Gene** | **Catalog Number** |
| --- | --- |
| R-Ras (HA-epitope) | RASR00TN00 |
| R-Ras Dominant Negative (HA-epitope) | RASR00TND0 |
| R-Ras (wild type) | RASR000000 |
| R-Ras2/TC21 (wild type) | TC21000000 |
